# TIM‐4 increases the proportion of CD4 ^+^ CD25 ^+^ FOXP3 ^+^ regulatory T cells in the pancreatic ductal adenocarcinoma microenvironment by inhibiting IL‐6 secretion

**DOI:** 10.1002/cam4.70110

**Published:** 2024-09-05

**Authors:** Ziyao Wang, Zerong Xie, Yu Mou, Ruiman Geng, Chen Chen, Nengwen Ke

**Affiliations:** ^1^ Division of Pancreatic Surgery, Department of General Surgery West China Hospital, Sichuan University Chengdu China; ^2^ Department of General Surgery West China Tianfu Hospital, Sichuan University Chengdu China; ^3^ Department of Biochemistry and Molecular Biology West China School of Basic Medical Sciences & Forensic Medicine, Sichuan University Chengdu China; ^4^ Department of Radiology The First People's Hospital of Chengdu Chengdu China

**Keywords:** IL‐6, PDAC, TIM‐4, Treg

## Abstract

**Background:**

Currently, creating more effector T cells and augmenting their functions is a focal point in pancreatic ductal adenocarcinoma (PDAC) treatment research. T cell immunoglobulin domain and mucin domain molecule 4 (TIM‐4), known for promoting cancer progression in various malignancies, is implicated in the suppressive immune microenvironment of tumors. Analyzing of the role of TIM‐4 in the immune regulation of PDAC can offer novel insights for immune therapy.

**Methods:**

We analyzed the TIM‐4 expression in tumor specimens from PDAC patients. Meanwhile, multiple fluorescent immunohistochemical staining was used to study the distribution characteristics of TIM‐4, and through tissue microarrays, we explored its correlation with patient prognosis. The influence of TIM‐4 overexpression on cell function was analyzed using RNA‐seq. Flow cytometry and ELISA were used for verification. Finally, the relationship between TIM‐4 and T lymphocytes was analyzed by tissue microarray, and the impacts of TIM‐4 on T cell subsets were observed by cell coculture technology and a mouse pancreatic cancer in situ model.

**Results:**

In PDAC, TIM‐4 is mainly expressed in tumor cells and negatively correlated with patient prognosis. TIM‐4 influences the differentiation of Treg by inhibiting IL‐6 secretion in pancreatic cancer cells and facilitates the proliferation of pancreatic cancer in mice. Additionally, the mechanism may be through the CD8^+^ effector T cells (CD8^+^Tc).

**Conclusion:**

TIM‐4 has the potential to be an immunotherapeutic target or to improve the efficacy of chemotherapy for PDAC.

## 1 INTRODUCTION

The 5‐year survival rate for pancreatic ductal adenocarcinoma (PDAC) is merely 10%, which is one of the lowest 5‐year survival rates of solid tumors.^1^ Current treatment is based on radical surgery and comprehensive chemotherapy, but even after completing the standard program, almost all patients eventually progress or relapse.^2^, ^3^ In recent years, various immunotherapies towards PD‐1 and PD‐L1, proven effective in another malignant tumor, have generally demonstrated inefficacy in PDAC.^4^ The immunosuppressive tumor microenvironment is found to be one of the causes of PDAC's “cold” response to immunotherapy.^5^ Regulatory T cells (Tregs), tumor‐associated macrophages (TAMs), marrow derived suppressor cells (MDSCs), and regulatory B cells (Bregs) are the major immunosuppressive cells in the PDAC microenvironment.^6^ Among them, Tregs inhibit the function of Teff (effector T cells) by influencing inhibitory cytokines (e.g., IL‐10, TGF‐β) or inhibitory receptors.^7^, ^8^, ^9^ The higher level of Treg infiltration in PDAC tumors, the lower overall survival of patients.^10^ It can be inferred that Tregs might serve as the strategy to achieve effective immunotherapy by influencing the aforementioned immune checkpoints.

TIM‐4 exhibited varied expression patterns in lung cancer, renal clear cell carcinoma, colorectal cancer and glioma, and closely related to tumor development and tumor immune microenvironment.^11^ Recent studies have found that TIM‐4^+^ macrophages can promote an immunosuppressive tumor state, and inhibiting TIM‐4 has been shown to substantially enhance the effectiveness of anti‐PD‐1 therapy in mouse peritoneal carcinoma models.^12^ Moreover, TIM‐4 binding to TIM‐3 on Th1 cell surfaces enhances p300 phosphorylation, leading to Th1 cell apoptosis. This suggests that TIM‐4, acting as a ligand for TIM‐3, may play a role in immune regulation.^13^ TIM‐3 is expressed on the membrane surface of Treg cells, and multiple animal clinical models have found that TIM‐3^+^ Tregs exhibit stronger immune inhibition than TIM‐3^−^Tregs.^14^, ^15^ In a word, reducing Treg levels through TIM‐4 blockade, a potential TIM‐3 ligand, is a novel approach to enhance anti‐PD‐1 therapy efficacy and improve the immunosuppressive microenvironment in PDAC.

Examining the expression and functional traits of TIM‐4 in PDAC, we attempted to enrich the content of the regulatory network of the immune microenvironment of PDAC and found evidence that TIM‐4 has the potential to be a target of immunotherapy to change the tumor microenvironment of PDAC.

## 2 MATERIALS AND METHODS

### 2.1 Cell lines and reagents

Cell lines (Panc1, MiaPaca‐2, Bxpc‐3, Panc02, and HPDE6‐C7) were sourced from the Chinese Academy Culture Collection (Shanghai, China) and cultured in DMEM or RPMI‐1640 medium with 10% FBS, 100 U/mL penicillin, and 100 μg/mL streptomycin at 37°C with 5% CO_2_.

### 2.2 Patients and clinical specimens

Thirty pairs of PDAC and corresponding normal pancreatic tissue samples were collected from the Department of Pancreatic Surgery of West China Hospital of Sichuan University from June 1, 2020, to May 30, 2021. These patients did not receive neoadjuvant chemotherapy or radiotherapy before surgery. Patient clinical information is listed in Table S1. Tumor and paracancerous tissues were defined as shown in Figure S1.

### 2.3 RNA extraction and qRT‐PCR

Total RNA was extracted using TriZol reagent (Invitrogen, California, USA), following the manufacturer's instructions. Reverse transcription was performed in a 10 μL system (2 μg total RNA) using the TIANGEN FastQuant RT Kit (Beijing, China). Real‐time PCR utilized the SYBR® Premix Taq kit (Takara Biotechnology, Shiga, Japan) with the recommended conditions. GAPDH served as the endogenous control, with primer sequences listed in Table S2. Relative gene expression levels were normalized to GAPDH using the 2^−ΔΔct^ method.

### 2.4 Western blot

Cells were lysed using RIPA buffer with 1% phenylmethylsulfonyl fluoride. Protein concentrations were quantified with the BCA Protein Quantification Kit (Vazyme, Nanjing, China) as per the manufacturer's instructions. Loading buffer was added to the samples and boiled for preparation. Protein samples were resolved on 10% SDS‐PAGE gels and transferred to NC membranes. After 1‐h blocking with 5% skimmed milk, membranes were incubated overnight with primary antibodies at 4°C. Antibodies and dilutions are listed in Table S3. After three washes with 0.1% TBST, secondary antibodies were added for 1 h at room temperature. Images were captured using a chemiluminescent substrate reagent (E411‐04, Vazyme, Nanjing, China) and an imaging instrument (ChemiDoc XRS, Bio‐Rad Laboratories, California, USA).

### 2.5 Multiplex fluorescent immunohistochemical staining and tissue imaging analysis

Paraffin‐embedded tissue sections from paired cancerous and paracancerous tissues were used for TMA construction and subjected to multiplex IHC analysis. Slides were deparaffinized, rehydrated, and underwent epitope retrieval in Tris‐EDTA buffer. Endogenous peroxidase was blocked with Antibody (Ab) Diluent/Block (72,424,205, PerkinElmer). After blocking, TMA slides underwent primary and secondary antibody labeling, visualized using the tyramide signal amplification (TSA) technique with an Opal™ 7‐color IHC Kit (NEL797B001KT, PerkinElmer). Antibodies used are listed in Table S4. Scanning was done with PerkinElmer Vectra (Vectra 3.0.5, PerkinElmer). Multispectral images were unmixed with inForm Advanced Image Analysis software (inForm 2.3.0, PerkinElmer). Mean fluorescence intensity (MFI) of each marker in each cell membrane was quantified, and positive cells were defined as those with a true immunofluorescence signal (> median MFI of all stained cells in a given slide) and the correct expression pattern.

### 2.6 Tissue microarrays

Tissue microarrays (HPanA150Su01) from 150 PDAC patients were obtained from Shanghai Outdo Biotech Co., Ltd. (Shanghai, China), and patient clinical details are listed in Table S5. For multiplex fluorescent immunohistochemical staining, the Opal™ 7‐color manual IHC kit (NEL801001KT, PerkinElmer, USA) was used according to the manufacturer's instructions. Antibodies used are listed in Table S4. Slide panoramic multispectral scanning was performed using the TissueFAXS system (TissueGnostics, Austria). Then, data were imported into StrataQuest analysis software, and the single channel fluorescence signal was obtained by spectral resolution using a spectral library. The tissue area, delineated by CK‐19, was statistically analyzed for cell counts in both tumor and interstitial regions.

### 2.7 Establishment of stable cell lines

Hanbio Biotechnology (Shanghai, China) provided the mouse full‐length TIM‐4 cDNA clone, and Panc02 cells were transduced with TIM‐4 recombinant lentivirus (Hanbio Biotechnology, Shanghai, China) to establish stable cell models. TIM‐4 sequences are in Table S6, and vector and target gene details are shown in Figure S2.

### 2.8 RNA‐seq and bioinformatics analysis

After resuscitation, total RNA was extracted from three kinds of cell lines. After confirming that the expression of TIM‐4 met the requirements, three biological repeats were prepared for each sample. Nine RNA samples were submitted to Shenzhen Huada Gene Science and Technology Service Co., Ltd (Shenzhen, China). DESeq software was utilized for gene count normalization and fold difference calculation. Differentially expressed genes were identified based on a fold difference >1.5 and *p* < 0.05. After sequencing, gene quantitative analysis, differential expression analysis and functional analysis were performed. The analysis process relied on the BGI Dr.tom system (Shenzhen, China).

### 2.9 TCGA database analysis

The data were downloaded from the TCGA website (www.cancer.gov). After data were downloaded, Perl software (Perl Foundation, USA) was used to convert the file into a matrix, download the corresponding gene files at the website (http://ensemblgenomes.org), and then convert all IDs in the matrix into identifiable gene names, and then use the R software V4.0.3 edgeR package to analyze the differentially expressed genes. The 178 pancreatic cancer samples in the TCGA database were downloaded from the Genomic Data Commons (GDC) data portal. A total of 332 normal tissue samples were collected from GTEx V8 (https://gtexportal.org/home/datasets) and analyzed statistically by R software V4.0.3. The Immunedeconv R package was used to perform immune scoring using R software V4.0.3.

### 2.10 ELISA

ELISA tests for IL‐6 and TGF‐β secreted from Panc02 cells were performed with a commercially available ELISA kit (RX20188 and RX20862, RUIXIN, Fujian, China) following the manufacturer's instructions. Protein concentrations, derived from standard curves, were expressed in pg/ml.

### 2.11 CCK‐8

Cell viability analysis involved co‐culturing tumor cells and lymphocytes in 96‐well plates for 12, 24, 36, and 48 h. Cells were then incubated in 10% Cell Counting Kit‐8 (CCK‐8; Dojindo Molecular Technologies, Inc., Kumamoto, Japan) solution, diluted in normal culture medium after lymphocyte washing, and observed for visual color conversion at 37°C. Absorbance in each well was measured at 450 nm using a VARIOSKAN FLASH (Thermo Fisher Scientific, Inc.).

### 2.12 Flow cytometry assay

Panc1, MiaPaca‐2, Bxpc‐3, HPDE6‐C7, and Panc02 cells were prepared, and corresponding direct flow antibodies for TIM‐4 were used to detect the expression characteristics of TIM‐4 in different cells. Panc02 (TIM‐4 overexpression, control, wild‐type, block) cell culture supernatant, cytokine standards and capture microspheres were prepared, cytokine detection was performed using a Mouse Th1/Th2/Th17 cytokine Kit (Cat.560485, BD Pharmingen, USA) and finally FCAP Array Software v3.0 (BD Pharmingen, USA) was used for subsequent data analysis. Antibodies and labels are listed in Table S7. Leuko Act Cktl With GolgiPlug (Cat. 550581, BD Pharmingen, USA) was used to costimulatory blockade. All flow cytometry data were acquired using the BD FACS Calibur cytometer (BD Biosciences, San Diego, CA, USA) and analyzed using FlowJo software (Treestar, Inc., San Carlos, CA, USA).

### 2.13 Cell coculture

Panc02 cells were prepared to applicable density, and then the single cell suspension was prepared. Transwell chambers (12 mm, 0.4 um PoreSize, Corning, USA) were used. The culture system consisted of 1 mL of completely fresh RPMI 1640 medium in the lower chamber and 500 μL of completely fresh RPMI 1640 medium in the upper chamber. The tumor cells of the four groups were cultured in the lower chamber with the same number (1 × 10^5^) for 12 h, and the block group added the blocking antibody (Anti‐mouse TIM‐4, Cat.BE0171, Bioxcell, USA) at 20 ug/ml. After cell stabilization, the prepared mouse lymphocytes (5 × 10^4^) were added to the upper chamber of each group and cultured in the same system for 24–36 h. After that, lymphocytes were collected for further experiments.

### 2.14 Animal studies

In this study, 6‐week‐old healthy male C57BL/JGpt mice with a body weight of 20 ± 1 g was used. All mice were raised in the Experimental Animal Center of West China Medical College of Sichuan University. Mouse lymphocytes were extracted using the animal spleen lymphocyte isolation kit (Cat.P8860, Solarbio, Beijing, China) according to the instructions, and then the mouse lymphocytes were amplified and cultured to an appropriate number for the next experiment. For mouse models of pancreatic cancer in situ, first, Panc02 cells were prepared to applicable density, and then the single cell suspension was prepared. A mixing matrix (Cat. 354248 Corning, Bedford, MA, USA) and cell suspension were mixed in a 1:1 ratio. The total volume was 600 μL containing 3 × 10^6^ cells, which were used in five mice. The block group was treated with intraperitoneal injection with the blocking antibody (Anti‐mouse TIM‐4, Cat.BE0171, Bioxcell, USA) 200 μg per mouse twice a week for 4 weeks. The mice were sacrificed after 30 days. The maximum diameter of the tumor was used as the basis for judging the tumor size. PD‐1 target medication came from Heng‐rui Co., Ltd (Cat. HRP00262‐022, Jiangsu, China).

### 2.15 Statistical analysis

Data are presented as mean ± standard deviation. Cut‐off values were determined using X‐tile software. Graphs were created with GraphPad Prism 8.0, and statistical analysis was conducted using SPSS 23.0 software. All experiments were replicated three times. Student's *t*‐test was used to compared means for normally distributed samples, the rank sum test was used for non‐normally distributed variables, and the chi‐square test was used for independent samples in the four‐cell table. *p* < 0.05 indicated statistical significance.

## 3 RESULTS

### 3.1 Expression characteristics of TIM‐4

**FIGURE 1:**
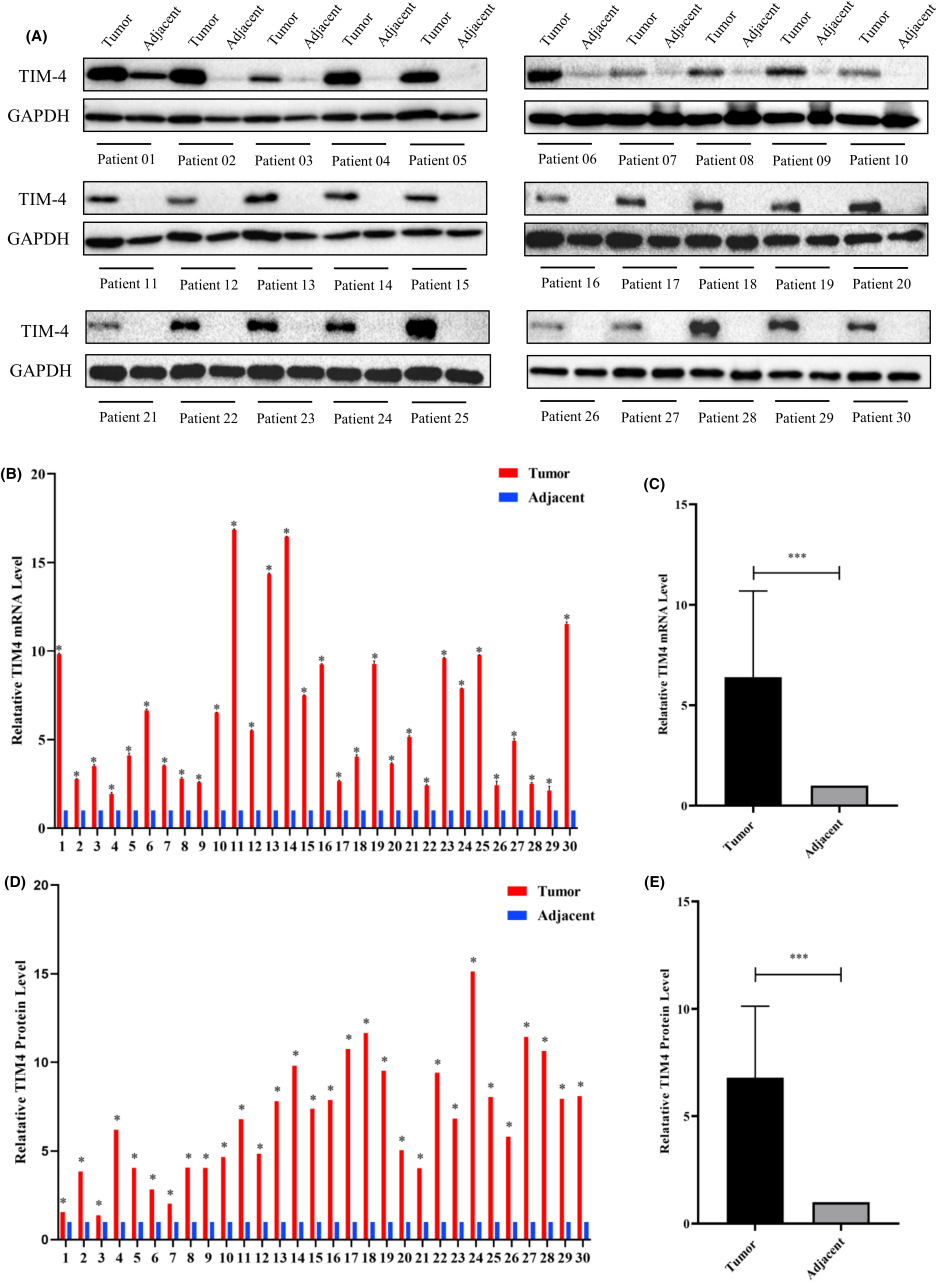
Expression characteristics of TIM‐4 in surgical excision specimens of PDAC patients. (A) TIM‐4 protein expression; (B, C) Statistical graph of TIM‐4 mRNA expression; (D, E) Statistical graph of TIM‐4 protein expression. *P < 0.05; ***P < 0.001.

**FIGURE 2:**
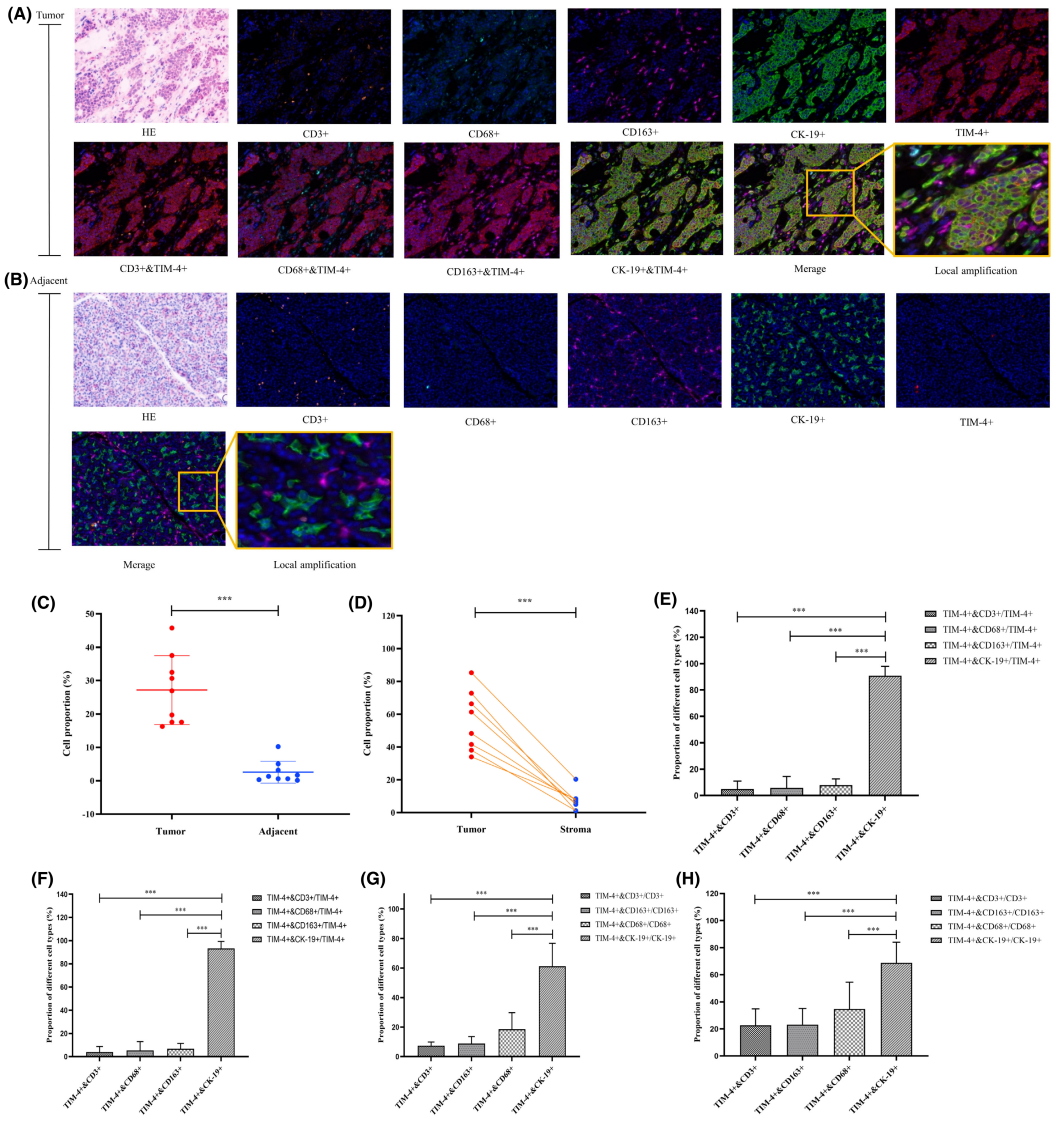
Expression characteristics and position of TIM‐4 in surgical excision specimens of PDAC patients. (A, B) HE staining and immunofluorescence histochemistry staining of typical PDAC cancer tissues and adjacent tissues of selected indexes; (C) Comparison of TIM‐4 expression in cancer tissues and adjacent tissues; (D) Comparison of TIM‐4 expression in tumor area and stroma area; (E) The proportion of different TIM‐4^+^ cells to all TIM‐4^+^ cells in cancer tissue; (F) The proportion of different TIM‐4^+^ cells in the tumor area of tumor area to all TIM‐4^+^ cells; (G) The proportion of various types of TIM‐4^+^ cells to that cell type in cancer tissues; (H) The proportion of TIM‐4^+^ cells in the tumor area to that cell type in tumor area; Red:TIM‐4^+^; Orange:CD3^+^ lymphocyte; Cyan:CD68^+^ macrophage; Pink:CD163^+^ macrophage; Green:CK‐19^+^; Blue: DAPI, ***P < 0.001.

Analyzing the TCGA database revealed elevated TIM‐4 expression in pancreatic cancer tissues compared to adjacent and normal pancreatic tissues. (Figure S3A,B). qPCR and Western Blot results indicated higher TIM‐4 expression in cancer tissues compared to adjacent tissues (Figure 1A–E). Results indicated significantly higher TIM‐4 expression in pancreatic cancer tissues compared to those in chronic pancreatitis and normal pancreatic tissues (Figure S4A–G). The composition of pancreatic cancer tumor tissues is complex, and includes tumor cells, immune‐related cells, fibroblasts, etc.^13^ According to previous studies, TIM‐4 is mainly expressed in macrophages, DCs, and tumor cells.^11^ Multicolor immunofluorescence histochemical staining was used to confirm the TIM‐4 expression in different cell types. Typical images of each index and co‐staining are shown in Figure 2A,B. Results revealed a higher proportion of TIM‐4^+^ cells in cancer tissues compared to paracancerous tissues (Figure 2C). After dividing the tumor tissue into tumor and stroma areas by software, it was found that the proportion of TIM‐4^+^ cells in tumor area was higher than that in stroma area (Figure 2D). Further analysis showed that the proportion of CK‐19^+^ cells in all TIM‐4^+^ cells of PDAC cancer tissue was higher than that of other cell types (Figure 2E). The proportion of CK‐19^+^ cells in all TIM‐4^+^ cells in the tumor cell area of cancer tissue was higher than that of other cell types (Figure 2F). These results suggest that the majority of TIM‐4 in PDAC expressed in tumor cells. Of course, the proportion of tumor cells in cancer tissues is higher than that of immune cells, so the proportion of TIM‐4^+^ cells in various cell types is also very important. According to the analysis, the ratio of TIM‐4^+^CK‐19^+^ cells to the CK‐19^+^ cells in the whole PDAC cancer tissue was significantly higher than other cell types (Figure 2G). the ratio of TIM‐4^+^CK‐19^+^ cells to the CK‐19^+^ cells in the tumor cell region of the whole cancer tissue was significantly higher than other cell types (Figure 2H). In conclusion, TIM‐4 mainly expressed in tumor cells in PDAC. Next, we found that the mRNA and protein expression levels of TIM‐4 were higher in all human pancreatic cancer cell lines than in human normal ductal epithelial cell lines (Figure S5A–C). Finally, TIM‐4 expressed in membrane and cytoplasm in all five cell lines by flow cytometry (Figure S5D–H), and the expression of TIM‐4 was higher in all human pancreatic cancer cell lines than in human normal ductal epithelial cell lines (Figure S5N,O). Meanwhile, the average fluorescence intensity of TIM‐4 in the four pancreatic cancer cell lines after membrane breaking staining was twice higher than that in the cell membrane staining only. Therefore, the expression of TIM‐4 in the cytoplasm was higher than that in the cell membrane in the four pancreatic cancer cell lines (Figure S5I–M).

### 3.2 Association between TIM‐4 expression and clinical characteristics of patients with PDAC

**FIGURE 3:**
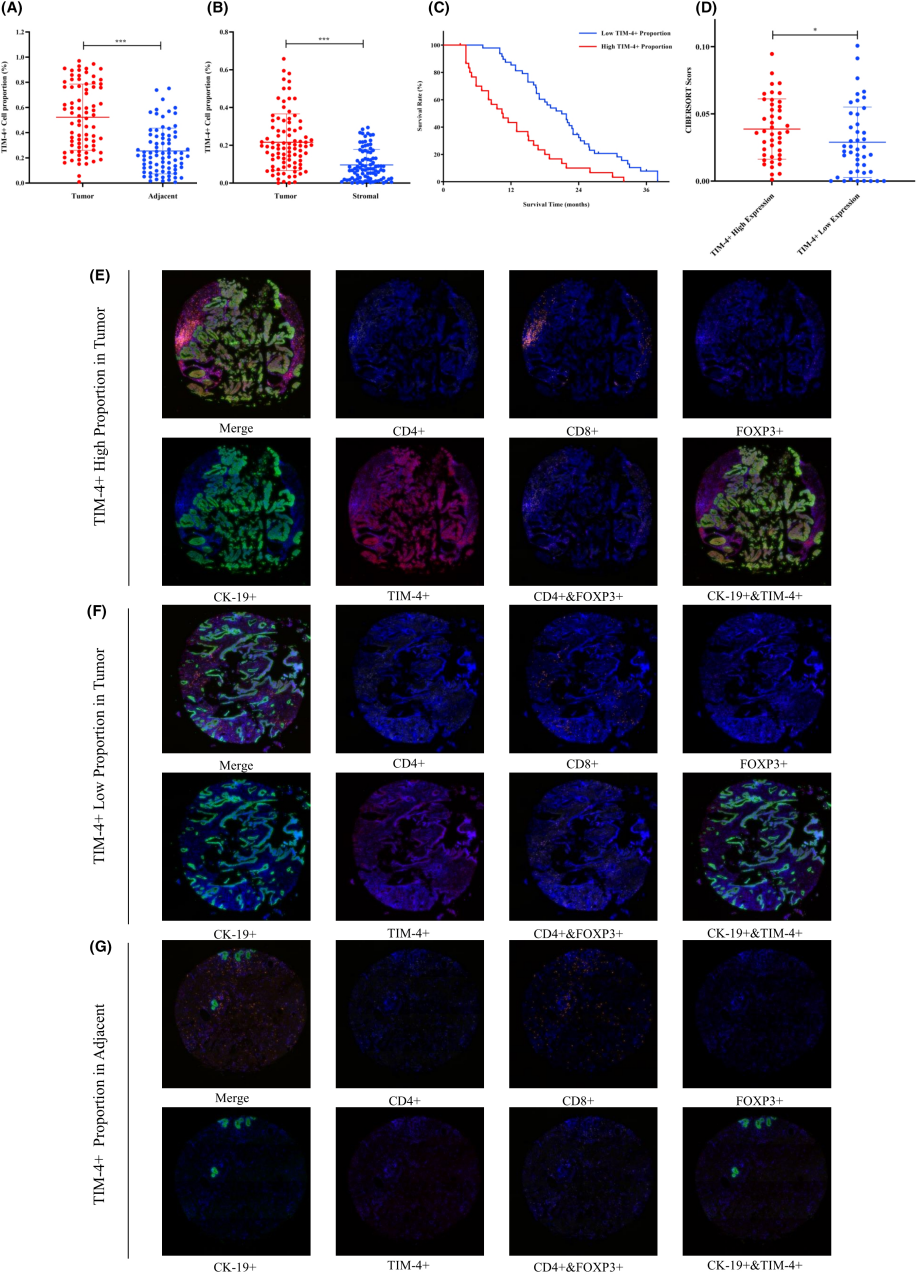
Expression and prognosis characteristics of TIM‐4 on tissue microarray chips in 79 patients with PDAC. (A) The proportion of TIM‐4^+^ cells in cancer tissues and adjacent tissues. (B) The proportion of TIM‐4^+^ cells in tumor area and stroma area in cancer tissue. (C) Survival time of PDAC patients with different proportion of TIM‐4^+^ cells. (D) Tregs immune scores of 88 pancreatic cancer patients with different TIM‐4 expression levels in the TCGA database. ***P < 0.001. (E–G) Representative immunofluorescence staining images of TIM‐4 high/low expression groups and adjacent tissues groups. Total magnification 200 × .

**FIGURE 4:**
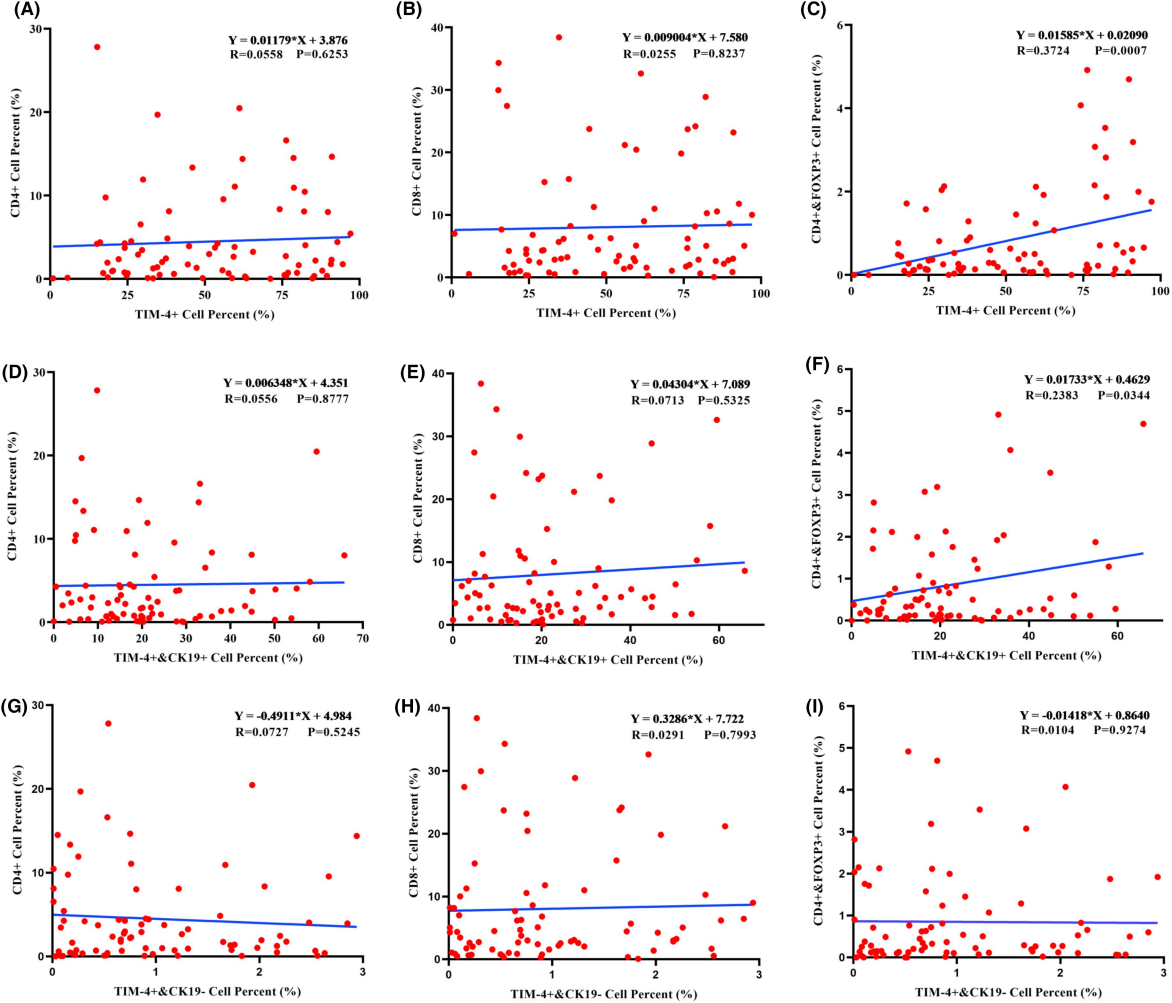
Relationship between TIM‐4^+^ cells and various types of lymphocytes. (A–C) The relationship between TIM‐4^+^ cells and CD4^+^/CD8^+^/CD4^+^FOXP3^+^ lymphocytes in cancer tissues; (D–F) The relationship between TIM‐4^+^ tumor cells and CD4^+^/CD8^+^/CD4^+^FOXP3^+^ lymphocytes; (G–I) The relationship between TIM‐4^+^ non‐tumor cells and CD4^+^/CD8^+^/CD4^+^FOXP3^+^ lymphocytes; R: Correlation Coefficient.

The effect of TIM‐4 on the clinical characteristics of patients was studied by tissue microarray chip. The result showed that the proportion of TIM‐4^+^ cells in cancer tissues was higher than that in adjacent tissues (Figure 3A), and the proportion of TIM‐4^+^ cells in the tumor area was higher in the cancer tissue than the stroma area (Figure 3B). Next, 79 samples were divided into TIM‐4 high/low expression group and clinical characteristics were analyzed. The results showed that TIM‐4 expression was not correlated with age, sex, differentiation, vascular invasion, nerve invasion, TNM stage, or tumor size (Table S5). In terms of prognosis, the survival time of patients with high TIM‐4 expression was significantly shorter than that of patients with low TIM‐4 expression (Figure 3C). Studies have indicated that the higher infiltration level of Tregs in PDAC, the worse overall survival time of patients.^10^ By studying the RNA‐seq data of 178 patients with pancreatic cancer in the TCGA database, the results showed that immune scores of Tregs in the TIM‐4 high expression group were higher than those in the low expression group. That is, patients with high TIM‐4 expression may have more Tregs in the tumor microenvironment (Figure 3D). Next, tissue microarray chips showed typical panoramic images of various indexes(Figure 3E–G), while Figure S6 shows a local magnification of typical images. By analyzing the proportion of different types of cells, we found that there was no linear relationship between the proportion of TIM‐4^+^ cells and CD4^+^/CD8^+^cells (Figure 4A,B). However, there was a significant linear correlation with the proportion of CD4^+^FOXP3^+^ Tregs (Figure 4C). To clarify the relationship between TIM‐4 on tumor cells and Tregs, CK‐19^+^TIM‐4^+^ cells were analyzed, and the results showed that there was no linear relationship between the proportion of CK‐19^+^TIM‐4^+^ cells and the CD4^+^/CD8^+^cells (Figure 4D,E). However, there was a linear relationship with the proportion of CD4^+^FOXP3^+^ Tregs (Figure 4F). Finally, we analyzed the relationship between TIM‐4 expression and lymphocytes in non‐tumor cells, and the results showed that there was no linear relationship between the proportion of CK‐19^−^TIM‐4^+^ cells and CD4^+^/CD8^+^/CD4^+^FOXP3^+^ cells (Figure 4G–I). Therefore, the higher expression of TIM‐4 on tumor cells may, by some mechanism, lead to an increased proportion of Tregs in the tumor microenvironment, leading to a poorer prognosis for patients.

### 3.3 The impact of high TIM‐4 expression on cellular functions in Panc02 cells

**FIGURE 5:**
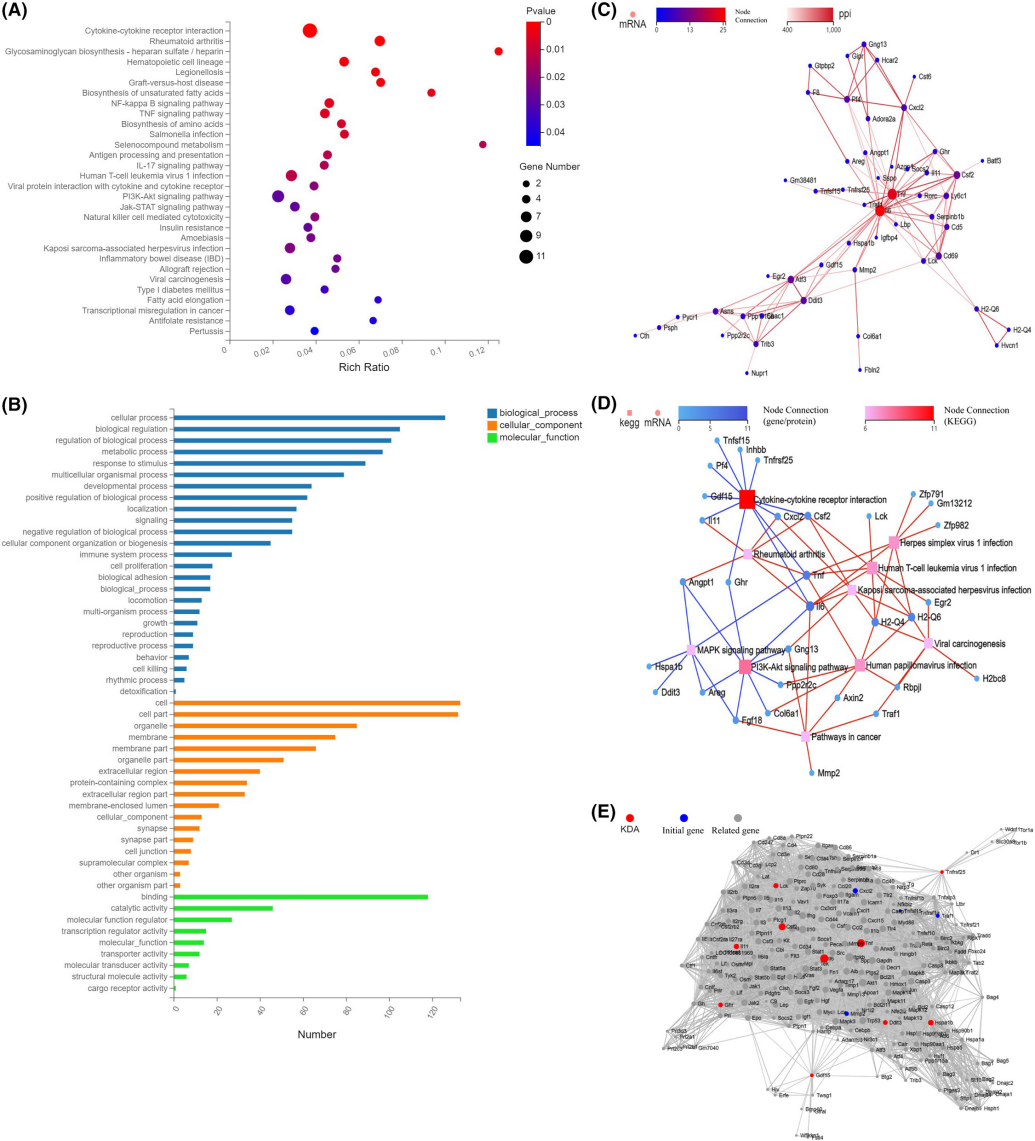
The effects of TIM‐4 overexpression on Panc02 cells were analyzed by RNA‐seq. (A) KEGG‐pathway enrichment analysis with significant differences. (B) GO enrichment analysis with significantly different. (C) 52 significantly different genes with PPI network interaction. (D) The KEGG pathway was performed in 52 significantly different genes with PPI network interaction. (E) Key driver genes in 52 significantly different genes with PPI network interaction.

First, the TIM‐4 overexpression Panc02 stable cell line and the empty control Panc02 cell line were constructed and verified (Figure S7). Next, RNA‐seq was performed in TIM‐4 overexpression group, Blank and Control group. First, the DEseq2 and VENN method was used to find the differentially expressed genes between the groups (Figure S8A,B). A total of 289 genes were found to be differentially expressed due to the overexpression of TIM‐4 (Figure S8C). Then, hierarchical cluster analysis was performed using heatmap. We selected the top 60 up‐regulated genes (Figure S9), and the top 100 down‐regulated genes for analysis (Figure S10). KEGG‐pathway analysis was performed on 160 genes, a total of 46 genes were mapped to 30 signaling pathways (*p* < 0.005) (Figure 5A; Table S8). At the same time, Gene Ontology (GO) enrichment analysis was applied to identify the biological attributes to complement the results of KEGG‐pathway analysis, and 154 significantly different genes were finally endowed with functional properties (Figure 5B). After GO analysis of significantly differentially expressed genes, protein–protein interaction (PPI) network analysis of these differentially expressed genes was carried out, and the network interaction diagram is shown in Figure 5C. The results revealed 52 genes in total. Among them, IL‐6 (node = 250) and TNF (node = 23) have the most connections and are at the core of the network diagram. After obtaining 52 differentially expressed genes with interactions, we used KEGG network interaction analysis to establish the KEGG network interaction diagram in Figure 5D, and the results showed that cytokine‐cytokine receptor interaction (node = 11) and PI3K‐Akt signaling pathway (node = 8) have the most interactions. IL‐6 is the only gene involved in both pathways. The analysis of PPI network and KEGG network suggested that IL‐6 plays a central role in this process. Next, based on the PPI network relationship, the key driver genes in the interaction network were analyzed, and the results showed that IL‐6 (node = 122) was the key driver gene (Figure 5E).

### 3.4 Effects of TIM‐4 on cytokine secretion in Panc02 cells

**FIGURE 6:**
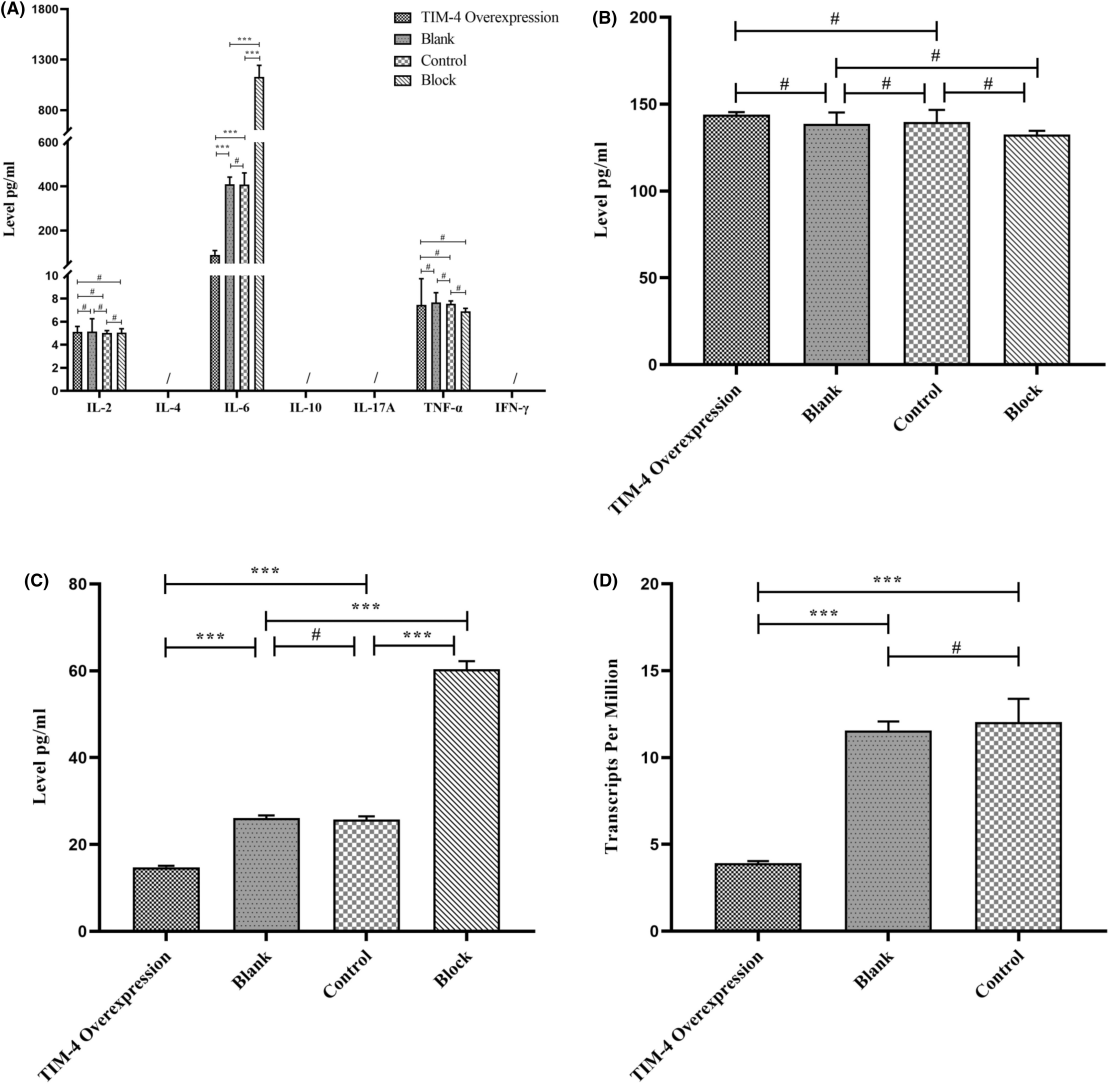
The different cytokine secretion in Panc02 stable cell lines. (A) The secretion levels of 7 cytokines in different TIM‐4 expression group; (B) TGF‐β secretion levels in different TIM‐4 expression group. (C) IL‐6 secretion levels among the four cell species by ELISA. (D) The expression level of IL‐6 mRNA in the three groups by RSEM. #: No significant difference; ***P < 0.001; /: Not detected.

Flow cytometry and ELISA were used to detect the cytokines secreted by TIM‐4 overexpressing Panc02 group, control group, and block group. The Flow cytometry results showed that IL‐2 was expressed in the four groups, but there was no significant difference among the them. IFN‐γ and IL‐4 were not detected in the four groups. In addition, IL‐10 and IL‐17A were not detected in four group. TNF‐α was detected in all four groups, but there was no statistical difference among them. IL‐6 was expressed in four groups, and the expression level in the TIM‐4 overexpression group was significantly lower than that of other groups (Figure 6A). For TGF‐β, which plays an important role in the differentiation of Th17 cells and Treg cells, we detected its expression in four groups by ELISA, but there was no significant difference among the four groups (Figure 6B). ELISA was used to verify the expression of IL‐6. The results showed that IL‐6 expression was significantly lower than that in the other groups (Figure 6C). Finally, RSEM was used to calculate the expression level of IL‐6 mRNA in TIM‐4 overexpression group, blank group and control group by analyzing the RNA‐seq,^16^ and the results showed that IL‐6 mRNA was expressed in all three groups, and the expression level of TIM‐4 overexpression group was significantly lower than that in other groups (Figure 6D). Based on the analysis of the above results, the effect of TIM‐4 on T lymphocyte subsets in Panc02 cells may be due to the differential secretion of IL‐6.

### 3.5 TIM‐4 overexpression induces the differentiation of regulatory T cells

**FIGURE 7:**
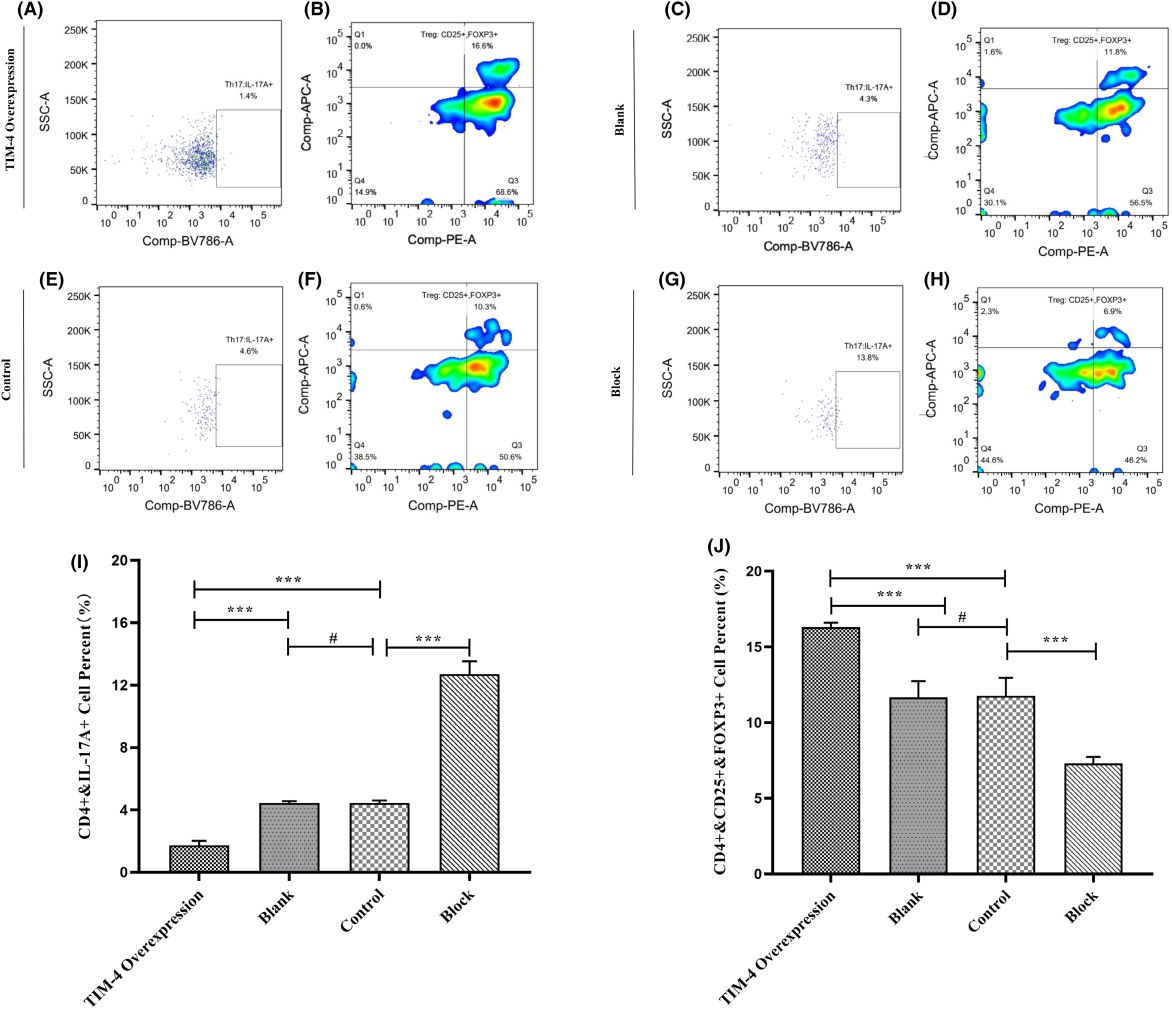
Flow cytometry was used to detect T lymphocyte subsets in different treatment groups. (A) Proportion of Th17 lymphocytes in TIM‐4 Overexpression group; (B) Proportion of Tregs in TIM‐4 Overexpression group; (C) Proportion of Th17 lymphocytes in Blank group; (D) Proportion of Tregs in Blank group; (E) Proportion of Th17 lymphocytes in Control group; (F) Proportion of Tregs in Control group; (G) Proportion of Th17 lymphocytes in Block group; (H) Proportion of Tregs in Block group; (I) Statistical results of TH17 cells in four different groups; (J) Statistical results of Treg cells in four different groups. #: No significant difference; ***P < 0.001.

In the pancreatic cancer microenvironment, Tregs account for almost 25% of the total CD4^+^T cells.^17^ IL‐6 inhibits the differentiation of naive CD4^+^T lymphocytes into FOXP3^+^ Tregs.^18^ The TIM‐4 overexpressing Panc02 cells, control Panc02 cells, and block TIM‐4 Panc02 cells were cocultured with T lymphocytes activated by CD3 and CD28. Statistical analysis was conducted after performing three independent experiments for each cohort. Flow cytometry was used to detect whether the differentiation of T cell subsets was affected by the TIM‐4 overexpression or blocking in vitro (Figure 7A–H; Figure S11A–H). Statistical analysis showed that there was no difference in the proportion of Th1/Th2 cells among the four groups (Figure S11I,J; Table S9). There was significant difference in the proportion of Th17 cells among the four groups (Figure 7I; Table S10). The proportion of Tregs were significantly higher in the TIM‐4 overexpressing Panc02 cell group than in the other three groups (Figure 7J; Table S10). In summary, TIM‐4 overexpression Panc02 cells induced CD4^+^Th cells to differentiate into CD4^+^CD25^+^FOXP3^+^ Tregs, but had no effect on Th1, Th2. Since Th17 cells and Treg cells are in a relatively balanced state during the differentiation, the number of Th17 cells will be relatively reduced when more CD4^+^ cells differentiate into Treg cells.

### 3.6 TIM‐4 overexpression leads to an increased proportion of Tregs in mouse pancreatic carcinoma in situ

**FIGURE 8:**
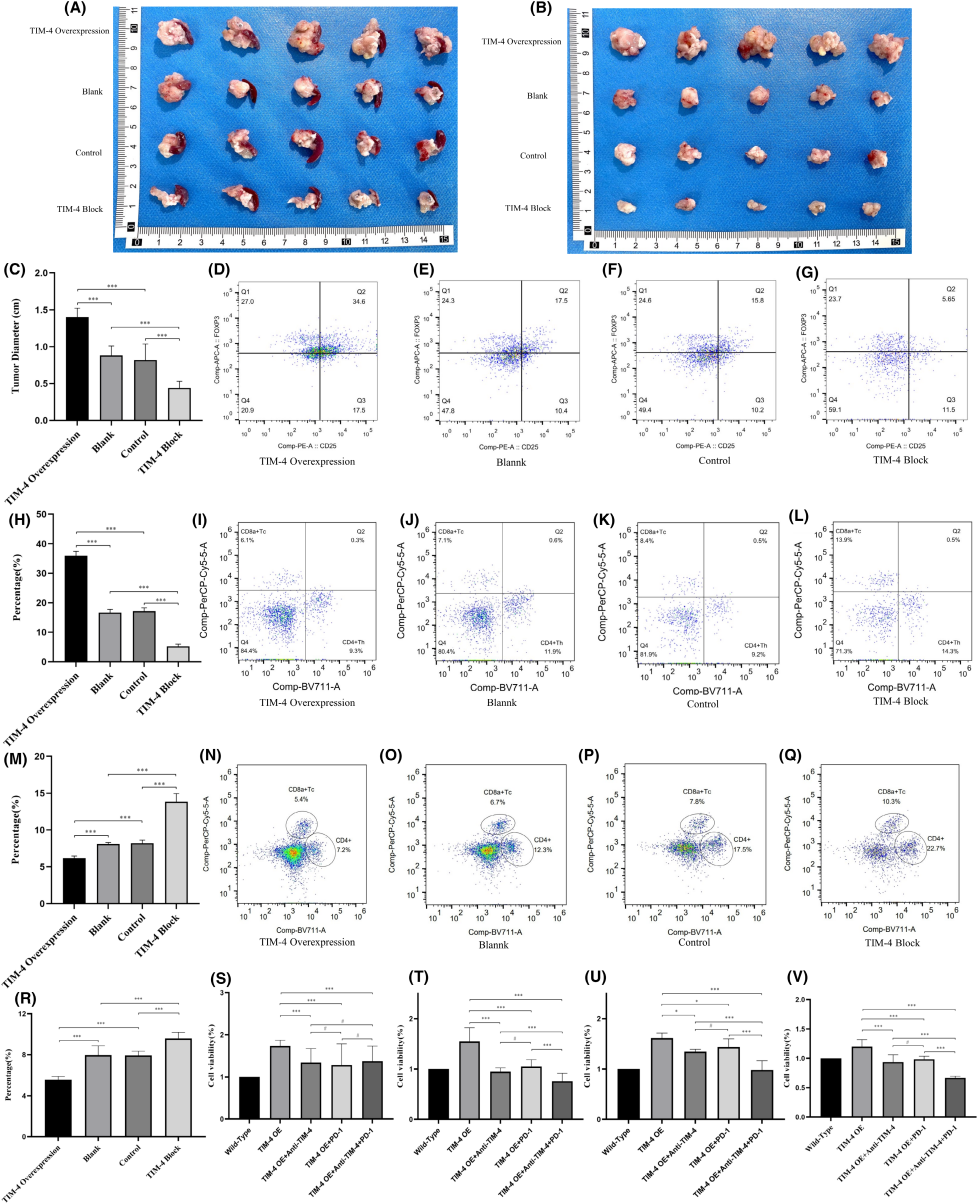
Mouse model of pancreatic cancer in situ and proportion of Tregs. (A) Pancreatic cancer in mouse of different groups, in situ display; (B) Pancreatic cancer in mouse of different groups, only tumors; (C) Tumor size in different groups, (D–G) Tregs in TIM‐4 overexpression group, Blank, Control and TIM‐4 block group; (H) Statistical analysis of the proportion of Tregs in four groups; (I–L) CD8^+^ Tc in TIM‐4 overexpression group, Blank, Control and TIM‐4 block group of mouse; (M) Statistical analysis of the proportion of CD8^+^ Tc in four groups of mouse; (N–Q) CD8^+^ Tc in TIM‐4 overexpression group, Blank, Control and TIM‐4 block group in vitro; (R) Statistical analysis of the proportion of CD8^+^ Tc in four groups in vitro. (S) Cell viability at 12 h after co‐culture; (T) Cell viability at 24 h after co‐culture; (U) Cell viability at 36 h after co‐culture; (V) Cell viability at 48 h after co‐culture. ***P < 0.001, #: No significant difference.

We used TIM‐4 overexpressing Panc02 cells, lentivirus empty Panc02 cells, wild‐type Panc02 cells and TIM‐4 blocking wild‐type Panc02 cells to construct an in‐situ tumor model of mouse pancreatic cancer. A total of 32 eligible mice were divided into four groups (TIM‐4 Overexpression, Blank, Control, Block), which received in situ tumor formation operation. A total of five mice in each group were included in the final analysis. The results showed that the tumor size of mice in the TIM‐4 overexpression group was larger than that in the other three groups (Figure 8A–C). Next, fresh anatomical tumor tissue was dissociated into single‐cell suspensions. Flow cytometry was used to detect the proportion of CD4^+^CD25^+^FOXP3^+^ Tregs in the four groups (Figure 8D–G). Data analysis revealed that the proportion of Tregs was higher in the TIM‐4 overexpression group than in the other groups (Figure 8H; Table S11). To further investigate the effect of TIM‐4 overexpression on the proportion of Treg cells, we measured the proportion of CD8^+^ effector T cells (CD8^+^Tc) in the above four groups (Figure 8I–L). The results showed that the proportion of CD8^+^Tc was significantly decreased in TIM‐4 overexpression group, while the proportion of CD8^+^Tc cells was significantly increased after TIM‐4 blockade (Figure 8M; Table S11). This trend was opposite to the proportion of Treg cells. This indicates that TIM‐4 overexpression leads to an increase in Treg cells, which eventually leads to a decrease in CD8^+^Tc and promotes tumor growth eventually. To further verify this result, we examined the proportion of CD8^+^Tc in the four groups in the in vitro environment **(**Figure 8N–Q **)**. The results showed that the number of CD8^+^Tc in TIM‐4 overexpression group was significantly lower than that in control group, while the number of CD8^+^Tc was significantly increased after TIM‐4 blockade(Figure 8R; Table S11). The poor efficacy of PD‐1 targeted therapy in PDAC is due to the suppressive immune microenvironment. Since blocking TIM‐4 could affect the proportion of Treg cells and CD8^+^Tc, we tried to verify whether blocking TIM‐4 and targeting PD‐1 could affect the growth of tumor cells in vitro. We tested the cell viability of wild‐type Panc02 cells, TIM‐4 overexpression group (OE), TIM‐4 OE + blocking TIM‐4 (Anti‐TIM‐4) group, TIM‐4 OE + PD‐1 monoclonal antibody(PD‐1) group, and TIM‐4 OE + Anti‐TIM‐4 + PD‐1 group under lymphocyte co‐culture conditions at 12, 24, 36, and 48 h by CCK‐8(Figure 8S–V). The results showed that TIM‐4 OE+ anti‐TIM‐4 + PD‐1 could significantly inhibit the growth of tumor cells caused by TIM‐4 overexpression at 48 h (Figure 8V). The inhibitory effect of Anti‐TIM‐4 + PD‐1 was significantly better than that of Anti‐TIM‐4 or PD‐1 antibody alone, moreover, the combined inhibitory effect of Anti‐TIM‐4 + PD‐1 made the cell viability of TIM‐4 OE + Anti‐TIM‐4 + PD‐1 group lower than that of Panc02 cells. Since the expression of TIM‐4 was significantly increased in PDAC cancer tissues, the above experiments simulated this situation, suggesting that combined targeting of TIM‐4 and PD‐1 may have a positive effect in inhibiting tumor cell growth.

## 4 DISCUSSION

It was initially thought that TIM‐4 was mainly expressed in APCs such as macrophages and mature DCs, further research revealed TIM‐4 expression in peritoneal B1 cells, mast cells, and iNKT cells..^19^, ^20^, ^21^ Moreover, TIM‐4 is expressed in various tumor cells, including lung cancer, kidney cancer, colorectal cancer, and glioma.^11^ In this study, we observed higher TIM‐4 expression in PDAC tumor tissues compared to adjacent tissues (Figure 1), mirroring the expression pattern observed in esophageal, colon, rectal, breast, and lung cancers.^22^ Further, identifying TIM‐4 expression localization in PDAC tissues is crucial, and the result showed that TIM‐4 in PDAC tumor tissues was predominant in tumor cells (Figure 2), consistent with observations in non‐small cell lung cancer tumor tissues.^23^ The classical view is that TIM‐4 is mainly involved in phagocytosis and recognition of apoptotic cells. However, even though TIM‐4 acts as a transmembrane protein on macrophages, TIM‐4 translocated into the cytoplasm after induced by inflammation, and promote the binding of STK11 and AMPKa in the cytoplasm to activate the autophagy signaling pathway in the local inflammatory environment.^24^, ^25^ Therefore, when acting in different roles in different cells, TIM‐4 not only functions as a membrane protein in signal transduction, but is often expressed in the cytoplasm, where it participates in various signaling pathways and the regulation of proteins. In this study, we detected that TIM‐4 expressed in both the cell membrane and cytoplasm in pancreatic cancer cell lines, but principally in the cytoplasm (Figure S5). Previous research has indicated a negative correlation between TIM‐4 and progression‐free survival in renal clear cell carcinoma patients,^26^ while high TIM‐4 expression is negatively correlated with overall survival in diffuse large B‐cell lymphoma, colorectal cancer, and lung cancer.^27^, ^28^, ^29^ In PDAC, our data showed that TIM‐4 was negatively correlated with overall survival time (Figure 3D). Due to the significant role of Tregs in the inhibitory immune microenvironment, we observed a positive correlation between TIM‐4 and Tregs cells in the tumor tissues of PDAC patients (Figure 4), and this phenomenon was also verified in malignant glioma.^30^ Our prior analysis of published PDAC single‐cell sequencing data revealed a tenfold increase in the number of Tregs in pancreatic cancer tissues compared to adjacent tissues,^31^, ^32^, ^33^ consistent with previous findings^34^, ^35^ (Figure S12).

TIM‐4 crucial role is recognizing apoptotic cells and regulating T cell function.^36^, ^37^ To fully understand the role of TIM‐4 in pancreatic cancer cells, we analyzed RNA‐seq data from TIM‐4 overexpressing Panc02 cells and their controls. Results indicated significant alterations in the PI3K‐Akt signaling pathway and the cytokine/cytokine receptor interaction pathway upon TIM‐4 overexpression. IL‐6, serving as the core driver gene, was implicated in both pathways (Figure 5). By analyzing the published single‐cell sequencing data of PDAC,^31^, ^32^, ^33^ we observed significantly lower IL‐6 expression in pancreatic cancer tissues compared to normal pancreatic tissues (Figure S13). For tumor cells, IL‐6 can drive the activation of STAT3, which can promote tumor initiation and progression, resist apoptosis and promote metastasis.^38^, ^39^, ^40^, ^41^ In addition to the above effects, in terms of immune regulation, IL‐6 inhibits differentiation of Treg induced by TGF‐β.^42^ The tumor microenvironment of PDAC is dominated by myeloid cell infiltration, but tumor cells could recruit immunosuppressive cells through cytokines, such as Tregs.^43^ These Tregs are usually adaptive Tregs that differentiate from initial CD4^+^T cells induced by tumor antigens and TGF‐β.^44^ We detected the changes in cytokine secretion in pancreatic cancer cells after TIM‐4 overexpression, the results demonstrated a notable reduction in IL‐6, hindering Treg differentiation (Figure 6). Subsequent cell coculture experiments also confirmed that overexpression of TIM‐4 promoted the differentiation of Treg (Figure 7). Since Treg and Th17 cell are in a state of dynamic balance during differentiation,^42^ we found that TIM‐4 overexpression promoted Treg differentiation, while the proportion of Th17 cells also decreased, thus inducing the tumor in an immunosuppressive state. Although overexpression of TIM‐4 leading to an increased proportion of Tregs was also observed in an in‐situ model of mouse pancreatic cancer (Figure 8), we cannot attribute the phenomenon in vivo solely to decreased IL‐6 secretion at present. In the tumor immune microenvironment, other cell types also secrete IL‐6, such as tumor‐associated macrophages and fibroblasts.^45^, ^46^, ^47^ In fact, as one of the most important sources of IL‐6, fibroblasts have unique spatial properties in the tumor microenvironment.^48^ This conclusion suggests that IL‐6 secretion may be spatially specific, even in the tumor microenvironment, cytokines in different regions have different expression levels.

In the mouse model of pancreatic cancer, TIM‐4 overexpression was observed to enhance tumor growth, while in vitro experiments showed no impact on tumor cell proliferation and apoptosis (Figure S14), indicating that the influence of TIM‐4 on tumors was more depending on the immune microenvironment. In vivo experimental, data indicated that TIM‐4 overexpression elevated the Treg proportion, while decreased the proportion of CD8^+^Tc cells, indicating that TIM‐4 affected CD8^+^Tc cells through Treg, and CD8^+^ Tc cells' killing effect on tumor cells ultimately affected tumor growth. Although targeting PD‐1/PD‐L1 therapies have recently developed rapidly in tumor treatment for various cancers, however, single targeting PD‐1/PD‐L1 therapy is not effective in pancreatic cancer.^49^ Studies indicate that alterations in the ratio of effector T cells, Tregs, MDSCs, and TAMs impact the response to anti‐PD‐1/PD‐L1 immunotherapy.^50^ Pancreatic cancer patients with high PD‐L1 levels may not respond to PD‐L1 therapy due to higher Tregs/MDSC/TAM infiltration.^49^ Combining IL‐6 targeting therapy with anti‐PD‐1/PD‐L1 can enhance T cell infiltration into tumors and alter the T cell phenotype within tumors.^50^ Tregs, effector T cells, and IL‐6, discussed in the preceding research, are all linked to TIM‐4 expression in this study. This suggests that altering TIM‐4 expression may impact the therapeutic effectiveness of PD‐1 in pancreatic cancer. Hence, we examined tumor cell survival using the combination of anti‐TIM‐4 and PD‐1 monoclonal antibody in vitro. The results indicated that targeting TIM‐4 could enhance the response of pancreatic cancer cells to PD‐1 monoclonal antibody (Figure 8). Of course, further in vivo experiments need to be verified.

In summary, we studied the expression characteristics of TIM‐4 in PDAC and its relationship with the clinical characteristics of patients, as well as its function. The current findings suggest that TIM‐4, a crucial node in the immune microenvironment regulatory network of PDAC, may significantly influence Treg differentiation. Although current studies of TIM‐4 are insufficient to identify it as a target for PDAC immunotherapy, due to its demonstrated immunomodulatory potential and possible interaction with the immune checkpoint target molecule, it shows research value in explaining the “cold” tumor microenvironment of PDAC and combination immune targeted therapy.

## AUTHOR CONTRIBUTIONS

**Ziyao Wang:** Project administration (lead); writing – original draft (lead). **Zerong Xie:** Data curation (equal); resources (equal). **Yu Mou:** Software (equal). **Ruiman Geng:** Formal analysis (equal). **Chen Chen:** Data curation (equal); methodology (equal). **Nengwen Ke:** Resources (lead); writing – review and editing (lead).

## FUNDING INFORMATION

This study was funded by West China Hospital Clinical Research Incubation Project (21HXFH058); the 1·3·5 Project for Disciplines of Excellence–Clinical Research Incubation Project (ZYJC21037), West China Hospital, Sichuan University.

## CONFLICT OF INTEREST STATEMENT

No conflict of interest.

## ETHICS STATEMENT

Human tumor specimens conformed to ethical standards and received approval from the Ethics Committee on Biomedical Research at West China Hospital of Sichuan University (No. 2015(267)). Informed consent was secured from all individual participants enrolled in the study.

## ANIMAL STUDIES

The Ethics Committee of West China Hospital, Sichuan University (No. 20211170A) approved this research.

## Supporting information


Figure S1.

## Data Availability

The data could be support by the corresponding author, upon reasonable request.

## References

[1] Mizrahi JD , Surana R , Valle JW , Shroff RT . Pancreatic cancer. Lancet. 2020;395:2008‐2020. 32593337 10.1016/S0140-6736(20)30974-0

[2] Conroy T , Desseigne F , Ychou M , et al. FOLFIRINOX versus gemcitabine for metastatic pancreatic cancer. N Engl J Med. 2011;364:1817‐1825. 21561347 10.1056/NEJMoa1011923

[3] Von Hoff DD , Ervin T , Arena FP , et al. Increased survival in pancreatic cancer with nab‐paclitaxel plus gemcitabine. N Engl J Med. 2013;369:1691‐1703. 24131140 10.1056/NEJMoa1304369 PMC4631139

[4] Bear AS , Vonderheide RH , Hara MH . Challenges and opportunities for pancreatic cancer immunotherapy. Cancer Cell. 2020;38:788‐802. 32946773 10.1016/j.ccell.2020.08.004 PMC7738380

[5] Gan LL , Hii LW , Wong SF , Leong CO , Mai CW . Molecular mechanisms and potential therapeutic reversal of pancreatic cancer‐induced immune evasion. Cancer. 2020;12:12. 10.3390/cancers12071872 PMC7408947 32664564

[6] Ware MB , El‐Rayes BF , Lesinski GB . Mirage or long‐awaited oasis: reinvigorating T‐cell responses in pancreatic cancer. J Immunother Cancer. 2020;8:8. 10.1136/jitc-2020-001100 PMC7449491 32843336

[7] Lee J , Su EW , Zhu C , et al. Phosphotyrosine‐dependent coupling of Tim‐3 to T‐cell receptor signaling pathways. Mol Cell Biol. 2011;31:3963‐3974. 21807895 10.1128/MCB.05297-11 PMC3187355

[8] Bauer CA , Kim EY , Marangoni F , Carrizosa E , Claudio NM , Mempel TR . Dynamic Treg interactions with intratumoral APCs promote local CTL dysfunction. J Clin Invest. 2014;124:2425‐2440. 24812664 10.1172/JCI66375 PMC4089459

[9] Noack M , Miossec P . Th17 and regulatory T cell balance in autoimmune and inflammatory diseases. Autoimmun Rev. 2014;13:668‐677. 24418308 10.1016/j.autrev.2013.12.004

[10] Orhan A , Vogelsang RP , Andersen MB , et al. The prognostic value of tumour‐infiltrating lymphocytes in pancreatic cancer: a systematic review and meta‐analysis. Eur J Cancer. 2020;132:71‐84. 32334338 10.1016/j.ejca.2020.03.013

[11] Wang Z , Chen C , Su Y , Ke N . Function and characteristics of TIM‐4 in immune regulation and disease. Int J Mol Med. 2023;51(2):10. 10.3892/ijmm.2022.5213 PMC9848438 36524355

[12] Chow A , Schad S , Green MD , et al. Tim‐4^+^ cavity‐resident macrophages impair anti‐tumor CD8^+^ T cell immunity. Cancer Cell. 2021;39:973‐988. 34115989 10.1016/j.ccell.2021.05.006 PMC9115604

[13] Huber M , Brehm CU , Gress TM , et al. The immune microenvironment in pancreatic cancer. Int J Mol Sci. 2020;21:19. 10.3390/ijms21197307 PMC7583843 33022971

[14] Sakuishi K , Ngiow SF , Sullivan JM , et al. TIM3^+^FOXP3^+^ regulatory T cells are tissue‐specific promoters of T‐cell dysfunction in cancer. Onco Targets Ther. 2013;2:e23849. 10.4161/onci.23849 PMC3654601 23734331

[15] Anderson AC . Tim‐3: an emerging target in the cancer immunotherapy landscape. Cancer Immunol Res. 2014;2:393‐398. 24795351 10.1158/2326-6066.CIR-14-0039

[16] Li B , Dewey CN . RSEM: accurate transcript quantification from RNA‐Seq data with or without a reference genome. BMC Bioinformatics. 2011;12:323. 21816040 10.1186/1471-2105-12-323 PMC3163565

[17] Saka D , Gökalp M , Piyade B , et al. Mechanisms of T‐cell exhaustion in pancreatic cancer. Cancer. 2020;12:12. 10.3390/cancers12082274 PMC7464444 32823814

[18] Korn T , Mitsdoerffer M , Croxford AL , et al. IL‐6 controls Th17 immunity in vivo by inhibiting the conversion of conventional T cells into Foxp^3+^ regulatory T cells. Proc Natl Acad Sci U S A. 2008;105:18460‐18465. 19015529 10.1073/pnas.0809850105 PMC2587589

[19] Wong K , Valdez PA , Tan C , Yeh S , Hongo JA , Ouyang W . Phosphatidylserine receptor Tim‐4 is essential for the maintenance of the homeostatic state of resident peritoneal macrophages. Proc Natl Acad Sci U S A. 2010;107:8712‐8717. 20421466 10.1073/pnas.0910929107 PMC2889355

[20] Zhang X , Gu J , Zhou L , Mi QS . TIM‐4 is expressed on invariant NKT cells but dispensable for their development and function. Oncotarget. 2016;7:71099‐71111. 27662666 10.18632/oncotarget.12153 PMC5340118

[21] Li L , Mo L , Hao H , et al. Flagellin modulates TIM4 expression in mast cells. Cell Biol Int. 2014;38:1330‐1336. 25044827 10.1002/cbin.10330

[22] Savill J , Dransfield I , Gregory C , Haslett C . A blast from the past: clearance of apoptotic cells regulates immune responses. Nat Rev Immunol. 2002;2:965‐975. 12461569 10.1038/nri957

[23] Liu W , Wang H , Bai F , et al. IL‐6 promotes metastasis of non‐small‐cell lung cancer by up‐regulating TIM‐4 via NF‐κB. Cell Prolif. 2020;53:e12776. 32020709 10.1111/cpr.12776 PMC7106962

[24] Shi CS , Shenderov K , Huang NN , et al. Activation of autophagy by inflammatory signals limits IL‐1β production by targeting ubiquitinated inflammasomes for destruction. Nat Immunol. 2012;13:255‐263. 22286270 10.1038/ni.2215 PMC4116819

[25] Liu W , Bai F , Wang H , et al. Tim‐4 inhibits NLRP3 Inflammasome via the LKB1/AMPKα pathway in macrophages. J Immunol. 2019;203:990‐1000. 31263038 10.4049/jimmunol.1900117

[26] Yano H , Motoshima T , Ma C , et al. The significance of TIMD4 expression in clear cell renal cell carcinoma. Med Mol Morphol. 2017;50:220‐226. 28631038 10.1007/s00795-017-0164-9

[27] Zhang Q , Wang H , Wu X , et al. TIM‐4 promotes the growth of non‐small‐cell lung cancer in a RGD motif‐dependent manner. Br J Cancer. 2015;113:1484‐1492. 26512878 10.1038/bjc.2015.323 PMC4815884

[28] Tan X , Zhang Z , Yao H , Shen L . Tim‐4 promotes the growth of colorectal cancer by activating angiogenesis and recruiting tumor‐associated macrophages via the PI3K/AKT/mTOR signaling pathway. Cancer Lett. 2018;436:119‐128. 30118845 10.1016/j.canlet.2018.08.012

[29] Li Y , Zhang PY , Yang ZW , Ma F , Li FX . TIMD4 exhibits regulatory capability on the proliferation and apoptosis of diffuse large B‐cell lymphoma cells via the Wnt/β‐catenin pathway. J Gene Med. 2020;22:e3186. 32187802 10.1002/jgm.3186

[30] Xu L , Xiao H , Xu M , Zhou C , Yi L , Liang H . Glioma‐derived T cell immunoglobulin‐ and mucin domain‐containing molecule‐4 (TIM4) contributes to tumor tolerance. J Biol Chem. 2011;286:36694‐36699. 21896488 10.1074/jbc.M111.292540 PMC3196134

[31] Peng J , Sun BF , Chen CY , et al. Single‐cell RNA‐seq highlights intra‐tumoral heterogeneity and malignant progression in pancreatic ductal adenocarcinoma. Cell Res. 2019;29:725‐738. 31273297 10.1038/s41422-019-0195-y PMC6796938

[32] Lin W , Noel P , Borazanci EH , et al. Single‐cell transcriptome analysis of tumor and stromal compartments of pancreatic ductal adenocarcinoma primary tumors and metastatic lesions. Genome Med. 2020;12:80. 32988401 10.1186/s13073-020-00776-9 PMC7523332

[33] Steele NG , Carpenter ES , Kemp SB , et al. Multimodal mapping of the tumor and peripheral blood immune landscape in human pancreatic cancer. Nat Cancer. 2020;1:1097‐1112. 34296197 10.1038/s43018-020-00121-4 PMC8294470

[34] Clark CE , Hingorani SR , Mick R , Combs C , Tuveson DA , Vonderheide RH . Dynamics of the immune reaction to pancreatic cancer from inception to invasion. Cancer Res. 2007;67:9518‐9527. 17909062 10.1158/0008-5472.CAN-07-0175

[35] Liyanage UK , Moore TT , Joo HG , et al. Prevalence of regulatory T cells is increased in peripheral blood and tumor microenvironment of patients with pancreas or breast adenocarcinoma. J Immunol. 2002;169:2756‐2761. 12193750 10.4049/jimmunol.169.5.2756

[36] Savill J , Gregory C . Apoptotic PS to phagocyte TIM‐4: eat me. Immunity. 2007;27:830‐832. 18093535 10.1016/j.immuni.2007.12.002

[37] Kuchroo VK , Dardalhon V , Xiao S , Anderson AC . New roles for TIM family members in immune regulation. Nat Rev Immunol. 2008;8:577‐580. 18617884 10.1038/nri2366

[38] van Duijneveldt G , Griffin M , Putoczki TL . Emerging roles for the IL‐6 family of cytokines in pancreatic cancer. Clin Sci. 2020;134:2091‐2115. 10.1042/CS20191211 PMC7434989 32808663

[39] Pop VV , Seicean A , Lupan I , Samasca G , Burz CC . IL‐6 roles—molecular pathway and clinical implication in pancreatic cancer—a systemic review. Immunol Lett. 2017;181:45‐50. 27876525 10.1016/j.imlet.2016.11.010

[40] Holmer R , Goumas FA , Waetzig GH , Rose‐John S , Kalthoff H . Interleukin‐6: a villain in the drama of pancreatic cancer development and progression. Hepatobiliary Pancreat Dis Int. 2014;13:371‐380. 25100121 10.1016/s1499-3872(14)60259-9

[41] Lesina M , Wörmann SM , Neuhöfer P , Song L , Algül H . Interleukin‐6 in inflammatory and malignant diseases of the pancreas. Semin Immunol. 2014;26:80‐87. 24572992 10.1016/j.smim.2014.01.002

[42] Kimura A , Kishimoto T . IL‐6: regulator of Treg/Th17 balance. Eur J Immunol. 2010;40:1830‐1835. 20583029 10.1002/eji.201040391

[43] Yao W , Maitra A , Ying H . Recent insights into the biology of pancreatic cancer. EBioMedicine. 2020;53:102655. 32139179 10.1016/j.ebiom.2020.102655 PMC7118569

[44] Chen W , Jin W , Hardegen N , et al. Conversion of peripheral CD4^+^CD25^−^ naive T cells to CD4^+^CD25^+^ regulatory T cells by TGF‐beta induction of transcription factor Foxp3. J Exp Med. 2003;198:1875‐1886. 14676299 10.1084/jem.20030152 PMC2194145

[45] Beatty GL , Chiorean EG , Fishman MP , et al. CD40 agonists alter tumor stroma and show efficacy against pancreatic carcinoma in mice and humans. Science. 2011;331:1612‐1616. 21436454 10.1126/science.1198443 PMC3406187

[46] Zhang Y , Yan W , Collins MA , et al. Interleukin‐6 is required for pancreatic cancer progression by promoting MAPK signaling activation and oxidative stress resistance. Cancer Res. 2013;73:6359‐6374. 24097820 10.1158/0008-5472.CAN-13-1558-T PMC3831882

[47] Lesina M , Kurkowski MU , Ludes K , et al. Stat3/Socs3 activation by IL‐6 transsignaling promotes progression of pancreatic intraepithelial neoplasia and development of pancreatic cancer. Cancer Cell. 2011;19:456‐469. 21481788 10.1016/j.ccr.2011.03.009

[48] Öhlund D , Handly‐Santana A , Biffi G , et al. Distinct populations of inflammatory fibroblasts and myofibroblasts in pancreatic cancer. J Exp Med. 2017;214:579‐596. 28232471 10.1084/jem.20162024 PMC5339682

[49] Feng M , Xiong G , Cao Z , et al. PD‐1/PD‐L1 and immunotherapy for pancreatic cancer. Cancer Lett. 2017;407:57‐65. 28826722 10.1016/j.canlet.2017.08.006

[50] Viehl CT , Moore TT , Liyanage UK , et al. Depletion of CD4^+^CD25^+^ regulatory T cells promotes a tumor‐specific immune response in pancreas cancer‐bearing mice. Ann Surg Oncol. 2006;13:1252‐1258. 16952047 10.1245/s10434-006-9015-y

