# Supplementary material for: TIM‐4 increases the proportion of CD4 + CD25 + FOXP3 + regulatory T cells in the pancreatic ductal adenocarcinoma microenvironment by inhibiting IL‐6 secretion
Source: Cancer Med. 2024 Sep 5;13(17):e70110. doi: 10.1002/cam4.70110 (PMC11375529; doi:10.1002/cam4.70110)
Supplement: Supplementary file 1 — Figure S1. [file CAM4-13-e70110-s001.docx]

**Supplement Figure/Table**


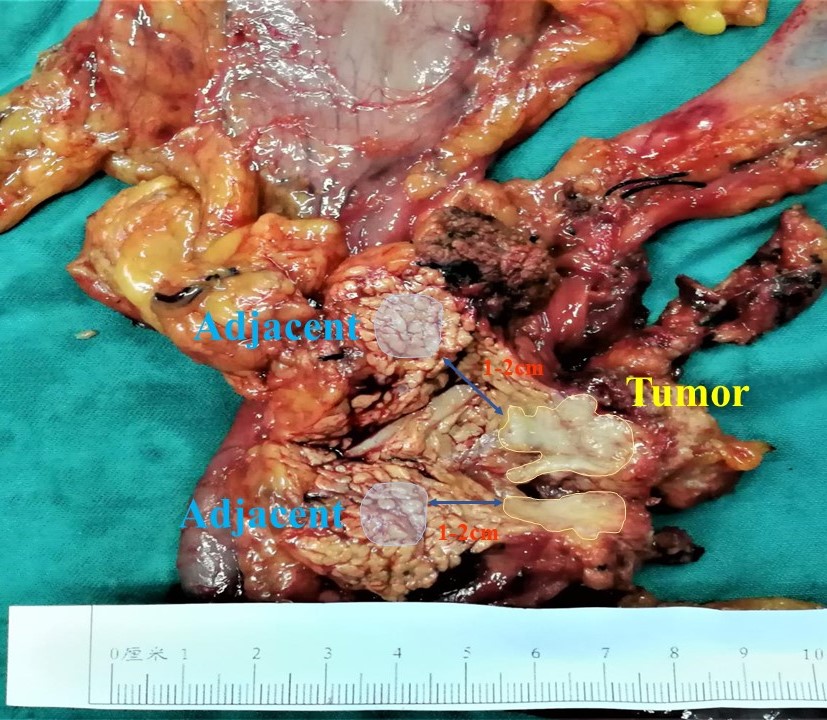


**Fig.S1.****The location of tumor tissue and paracancerous tissue**


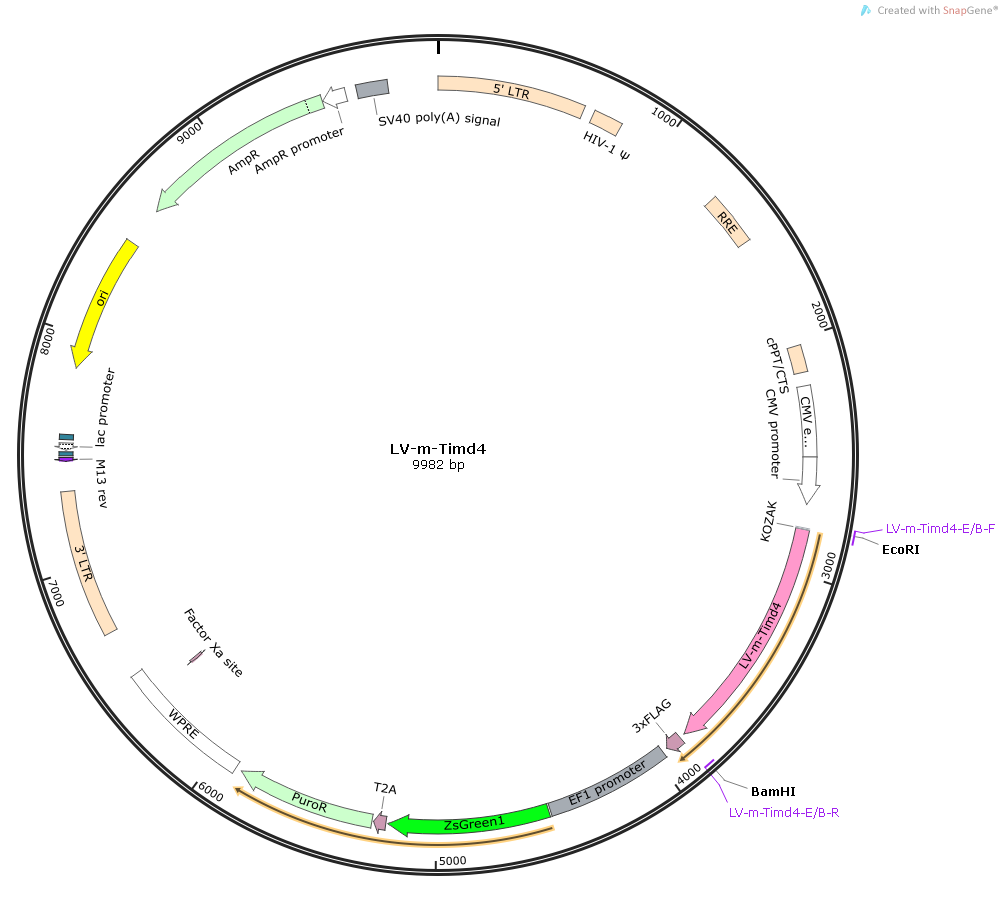
 **Fig.S2. Diagram of expression vector: pHBLV-CMV-MCS-3FLAG-EF1-ZsGreen-T2A-PURO**


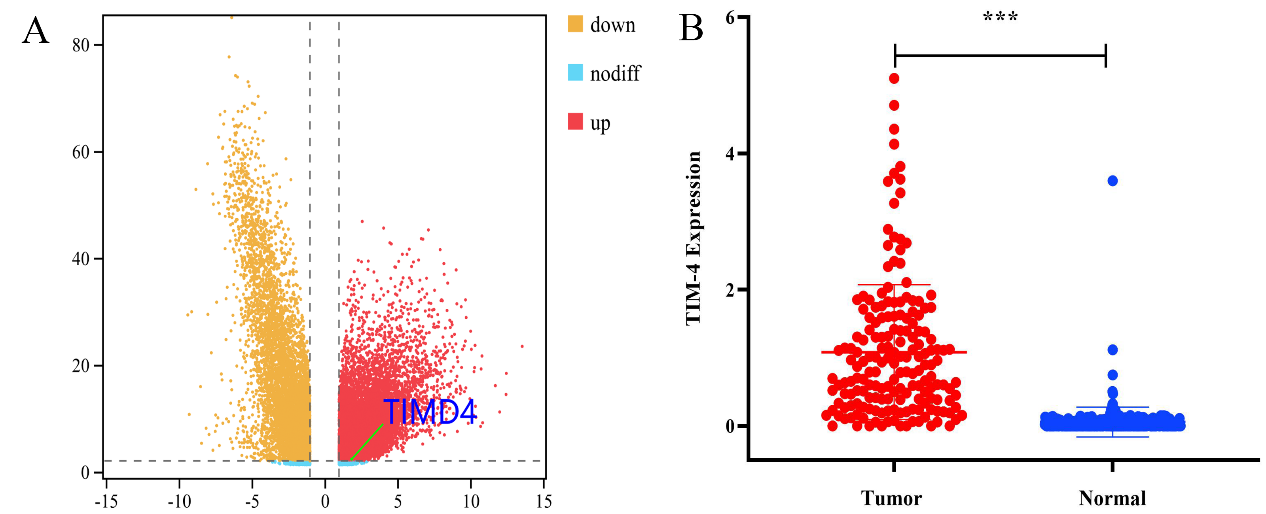


**Fig.S3. The expression characteristics of TIM-4 analyzed by TCGA database**

A: Differential gene analysis of 30 pairs of pancreatic cancer tissues and corresponding adjacent tissues in TCGA database. Volcano plots are created using fold change and adjusted p-values. red dots :upregulated, yellow dots: downregulated, and blue dots: not significant. B:Expression difference of TIMD4 in 178 cases of pancreatic tumor and 332 cases of normal pancreatic tissue in TCGA database. ***：P<0.001


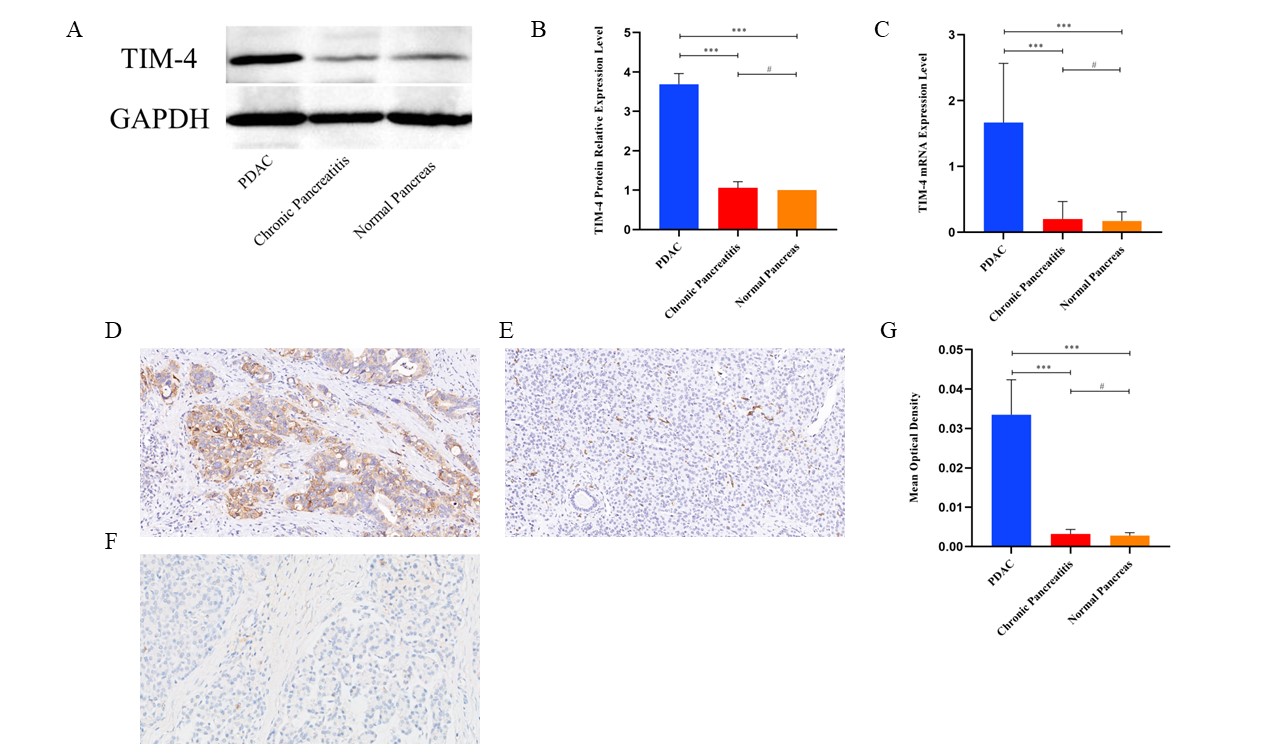


**Fig.S4.** **Expression characteristics of TIM-4 in different tissue**

A: Western Blot images of TIM-4 expression in PDAC tissues, chronic pancreatitis tissues, and normal pancreatic tissues; B: Statistical graph of TIM-4 expression in PDAC tissues, chronic pancreatitis tissues, and normal pancreatic tissues; C: Statistical graph of TIM-4 mRNA expression in PDAC tissues, chronic pancreatitis tissues, and normal pancreatic tissues; D: Immunohistochemical staining of TIM-4 expression in PDAC tissues; E: Immunohistochemical staining of TIM-4 expression in normal pancreatic tissue; F: Immunohistochemical staining of TIM-4 expression in chronic pancreatitis tissue; G: Mean optical density values of immunohistochemical staining for TIM-4 expression in PDAC tissue, chronic pancreatitis tissue, and normal pancreatic tissue. *** : P<0.001; #:P>0.05; Total magnification 200×.


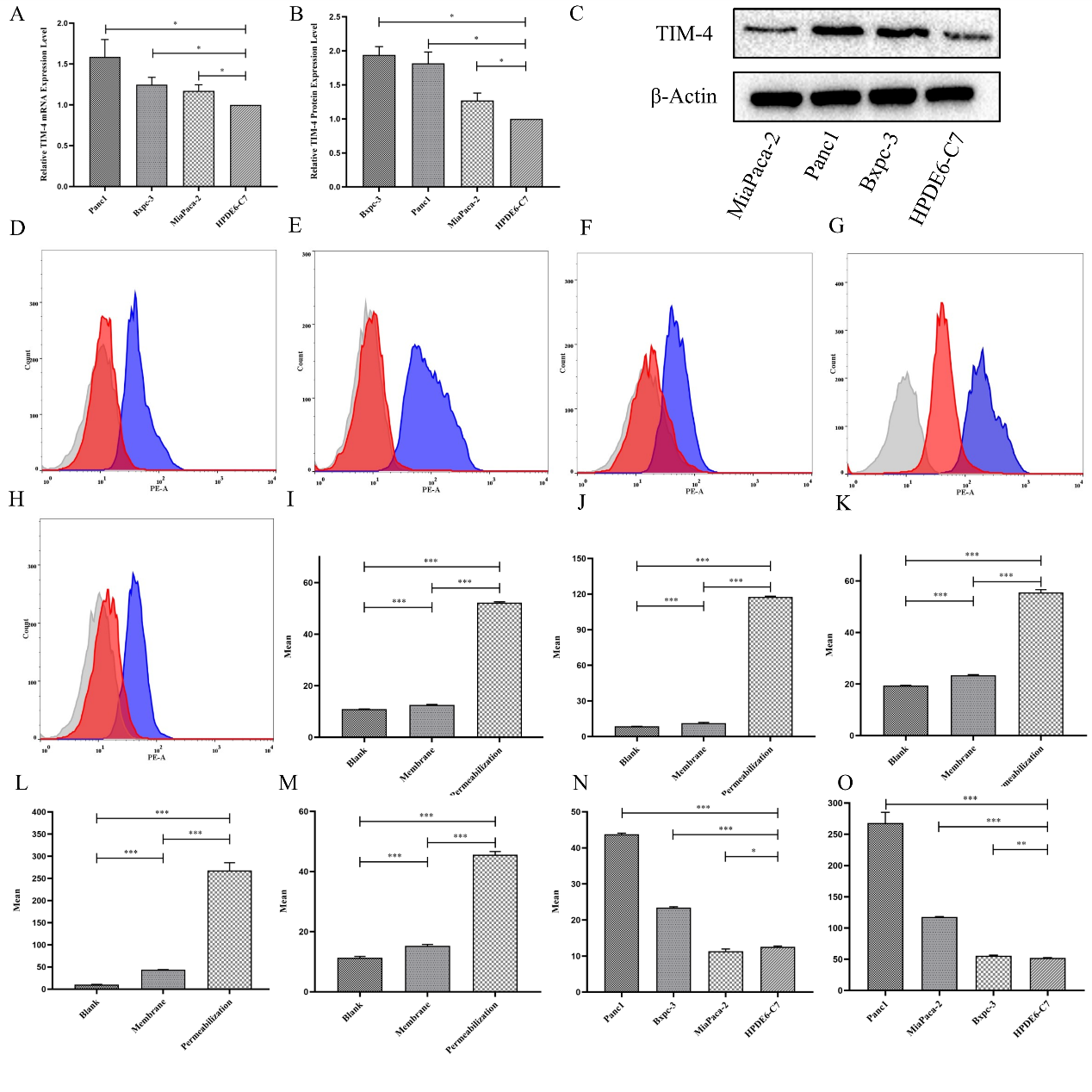


**Fig.S5.** **Expression characteristics of TIM-4 in human/mouse pancreatic cancer cell lines and human normal pancreatic ductal epithelial cell lines**

A: Expression levels of TIM-4 mRNA in human pancreatic cancer cell lines and normal pancreatic ductal epithelial cell lines, B: Protein expression levels of TIM-4 in human pancreatic cancer cell lines and normal pancreatic ductal epithelial cell lines, C: Western Blot analysis of TIM-4 in human pancreatic cancer cell lines and normal pancreatic ductal epithelial cell lines, D: The expression characteristics of TIM-4 in HPDE6-C7 were detected by flow cytometry. E-G: Flow cytometry was used to detect the expression characteristics of TIM-4 in MiaPaca-2, Bxpc-3 and Panc1, H: The expression characteristics of TIM-4 in Panc02 were detected by flow cytometry, I-M: Comparison of TIM-4 expression levels in HPDE6-C7, MiaPaca-2, Bxpc-3, Panc1, Panc02 cells, N: Comparison of cell membrane TIM-4 expression in human pancreatic cancer cell lines and normal pancreatic ductal epithelial cell lines, O: Comparison of total TIM-4 expression in human pancreatic cancer cell lines and normal pancreatic ductal epithelial cell lines. Gray: Blank group, not dyed; Red: Membrane group, which only stains the membrane; Blue: Permeabilization group, staining both cell membrane and cytoplasm, Panc1, MiaPaca-2, Bxpc-3: human pancreatic cancer cell line; HPDE6-C7: human normal pancreatic ductal epithelial cell line, *:P<0.05,*** : P<0.001.


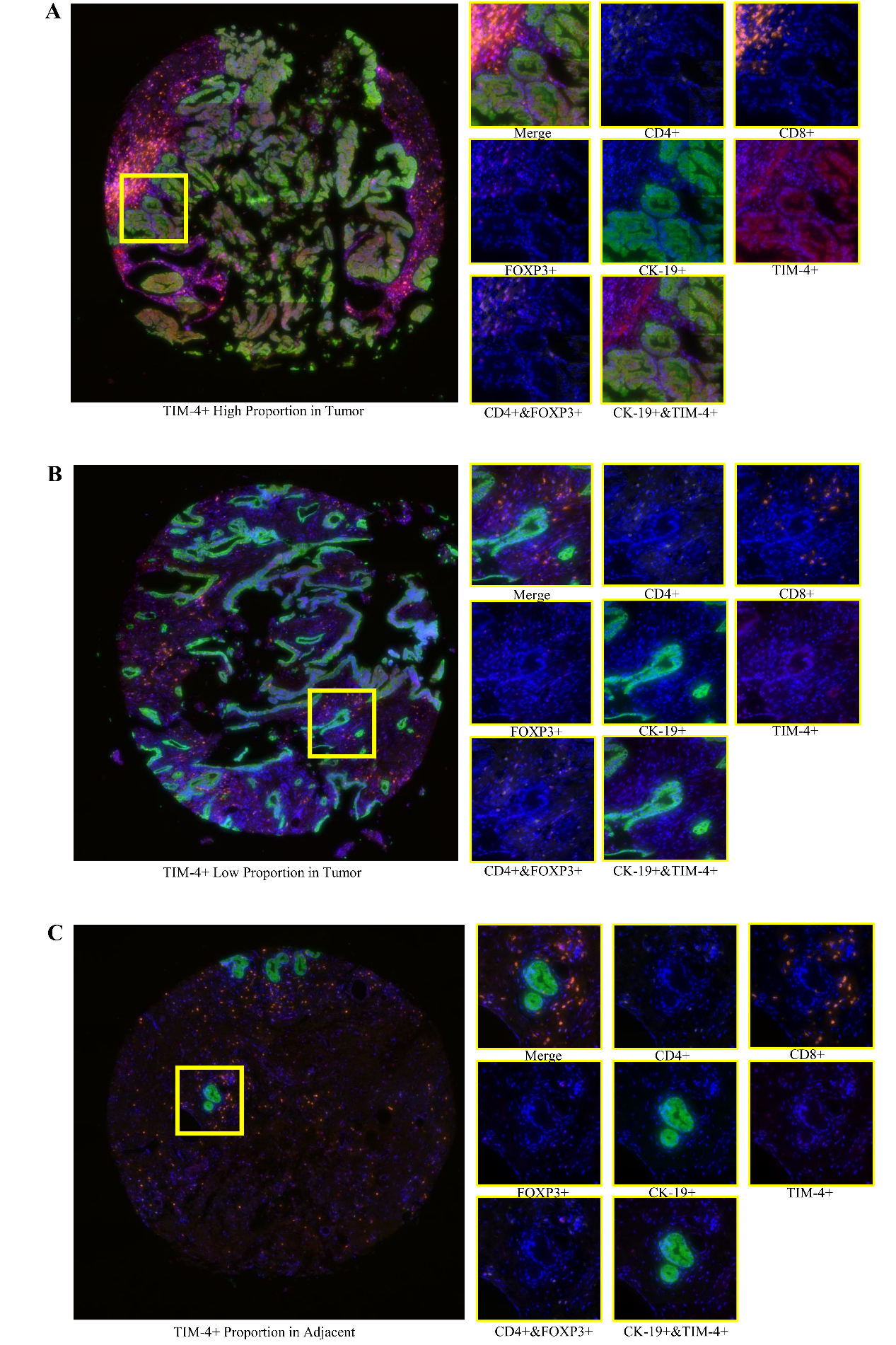


**Fig.S6.** **Local images of indicators of TIM-4 high/low expression group and adjacent tissues**

A: TIM-4 high expression group, B: TIM-4 low expression group, C: adjacent tissue group, Merge: multicolor mixed image, CD4^+^ : yellow, CD8^+^: orange, FOXP3: pink, CK-19: Green, TIM-4: red, DAPI: Blue. Total magnification 200×.
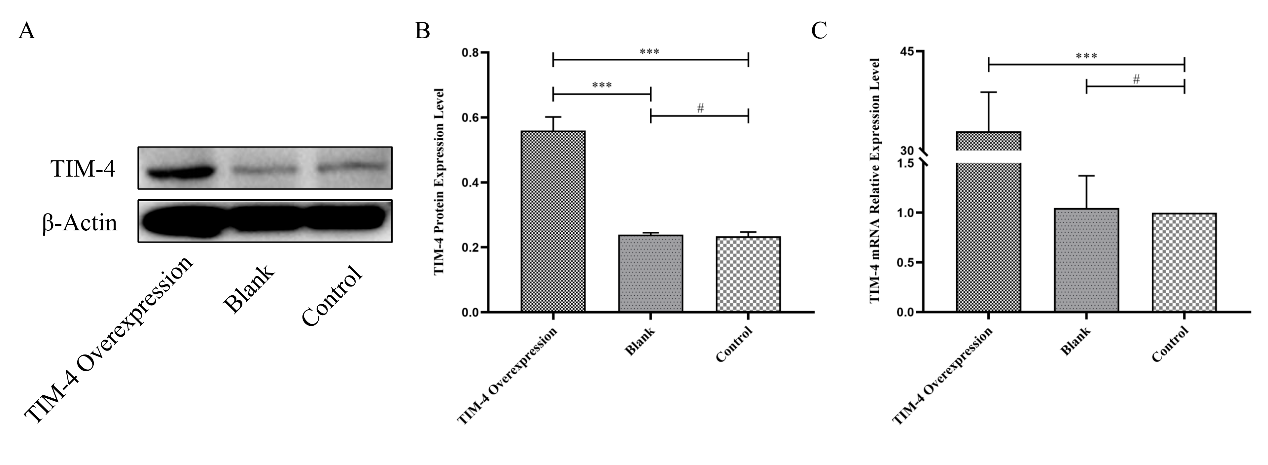


**Fig.S7. Validation of transfection effect of Panc02 TIM-4 overexpression stable cell line**

A: Western Blot showed the expression of TIM-4 in Panc02 TIM-4 overexpression stable cell line and its control group. B: Statistical graph of TIM-4 protein expression in Panc02 TIM-4 overexpression stable cell line and its control group, C: Statistical graph of TIM-4 mRNA expression in Panc02 TIM-4 overexpression stable cell line and its control group, TIM-4 Overexpression: Panc02 TIM-4 overexpression stable cell line, Blank: lentivirus-empty Panc02 stable cell line; Control: wild-type Panc02 cell, *** : P<0.001, # : nonsignificant difference.


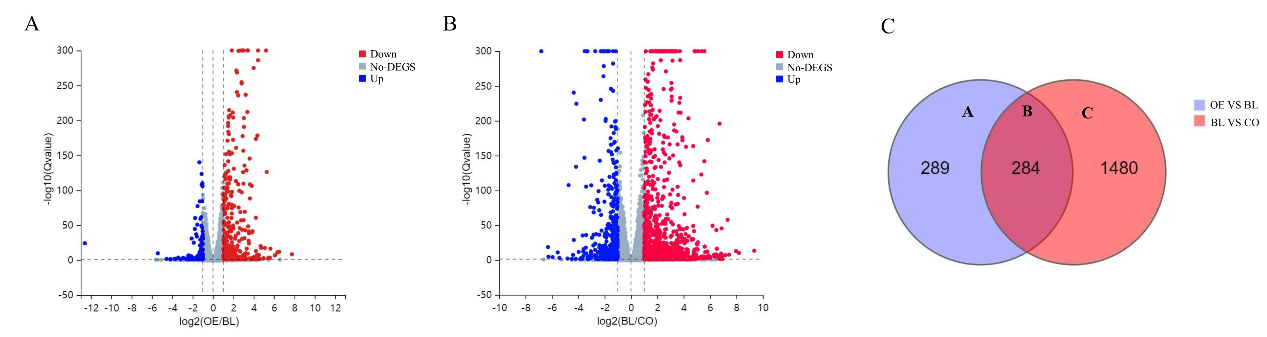


**Fig.S8. Differential gene analysis in Panc02 TIM-4 overexpression stable cell line and its control group**

A: Differential gene of TIM-4 overexpression group compared with no-load group, B: Differential genes of no-load group compared with control group; C: VENN analysis of differential genes in 3 groups. OE: Panc02 TIM-4 overexpression group; BL: Blank, lentivirus no-load group; CO: Control, wild-type Panc02 cell, No-DEGS: No difference.


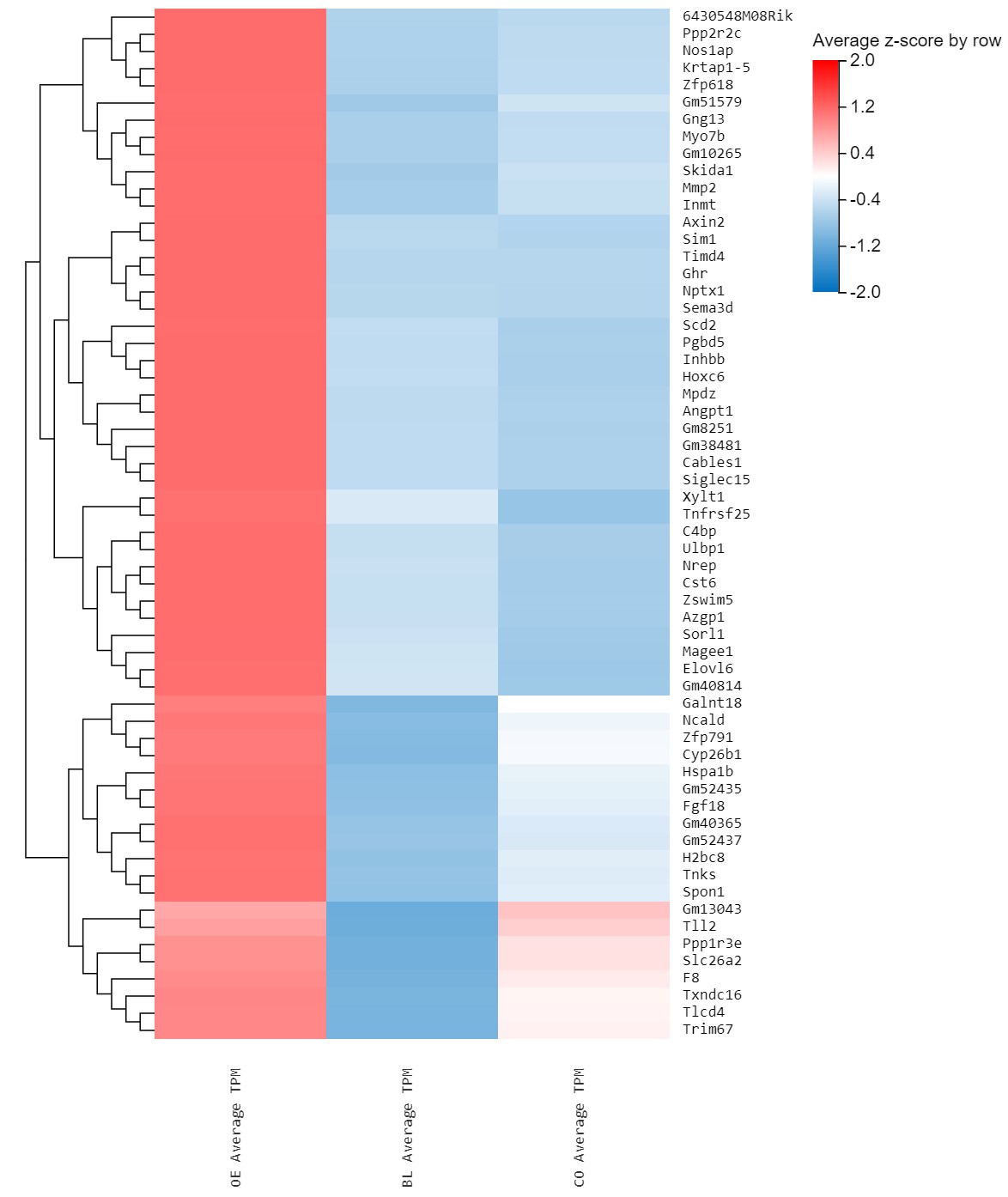


**Fig.S9. Clustering analysis of expression levels of the first 60 up-regulated differential genes in Panc02 TIM-4 overexpression stable cell line and its control group**

OE: Panc02 TIM-4 overexpression group, BL: Blank, lentivirus no-load group, CO: Control, wild-type Panc02 cell, TPM: Transcripts Per Kilobase Million.


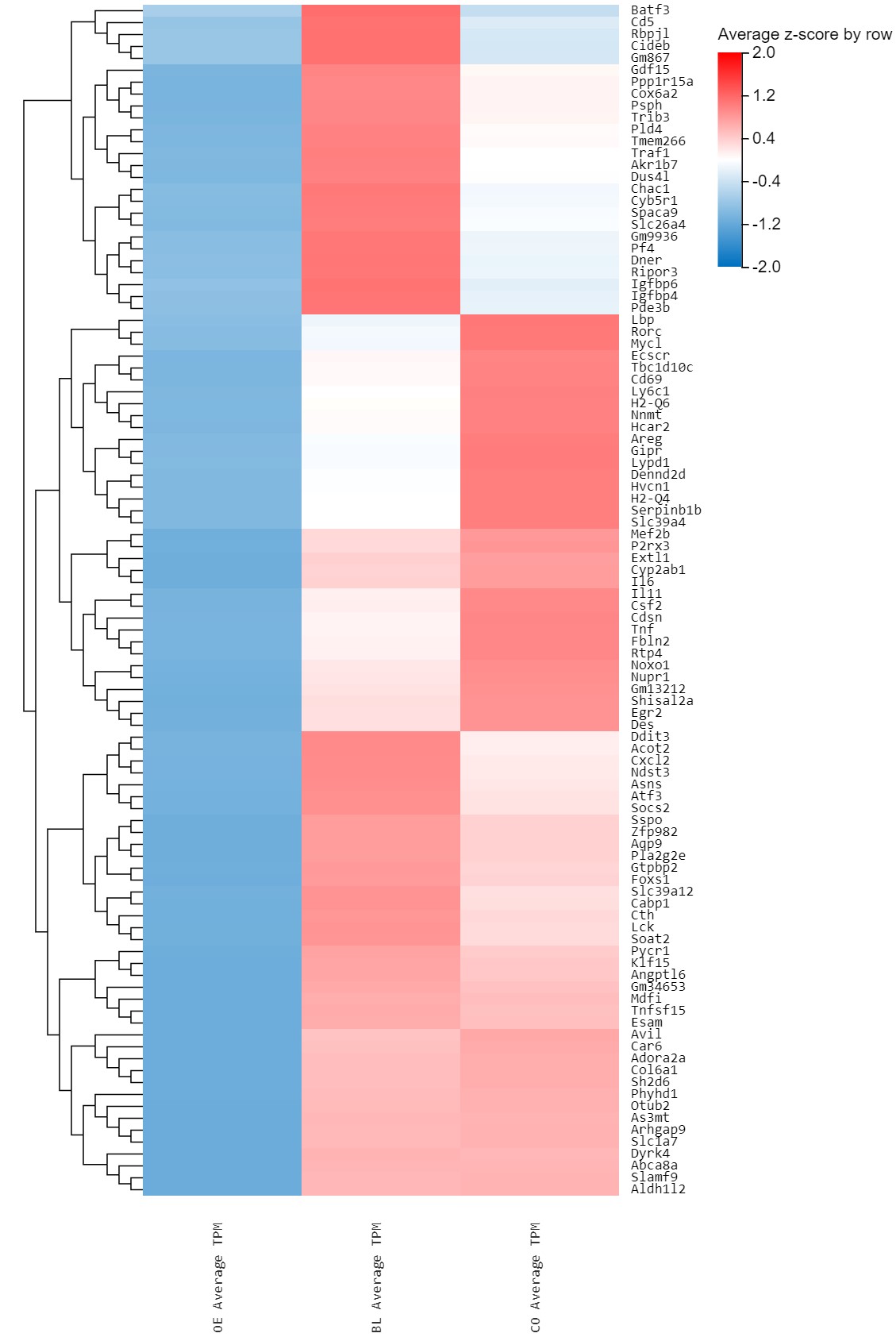


**Fig.S10. Clustering analysis of expression levels of the first 100 down-regulated differential genes in Panc02 TIM-4 overexpression stable cell line and its control group**

OE: Panc02 TIM-4 overexpression group, BL: Blank, lentivirus no-load group, CO: Control, wild-type Panc02 cell, TPM: Transcripts Per Kilobase Million.


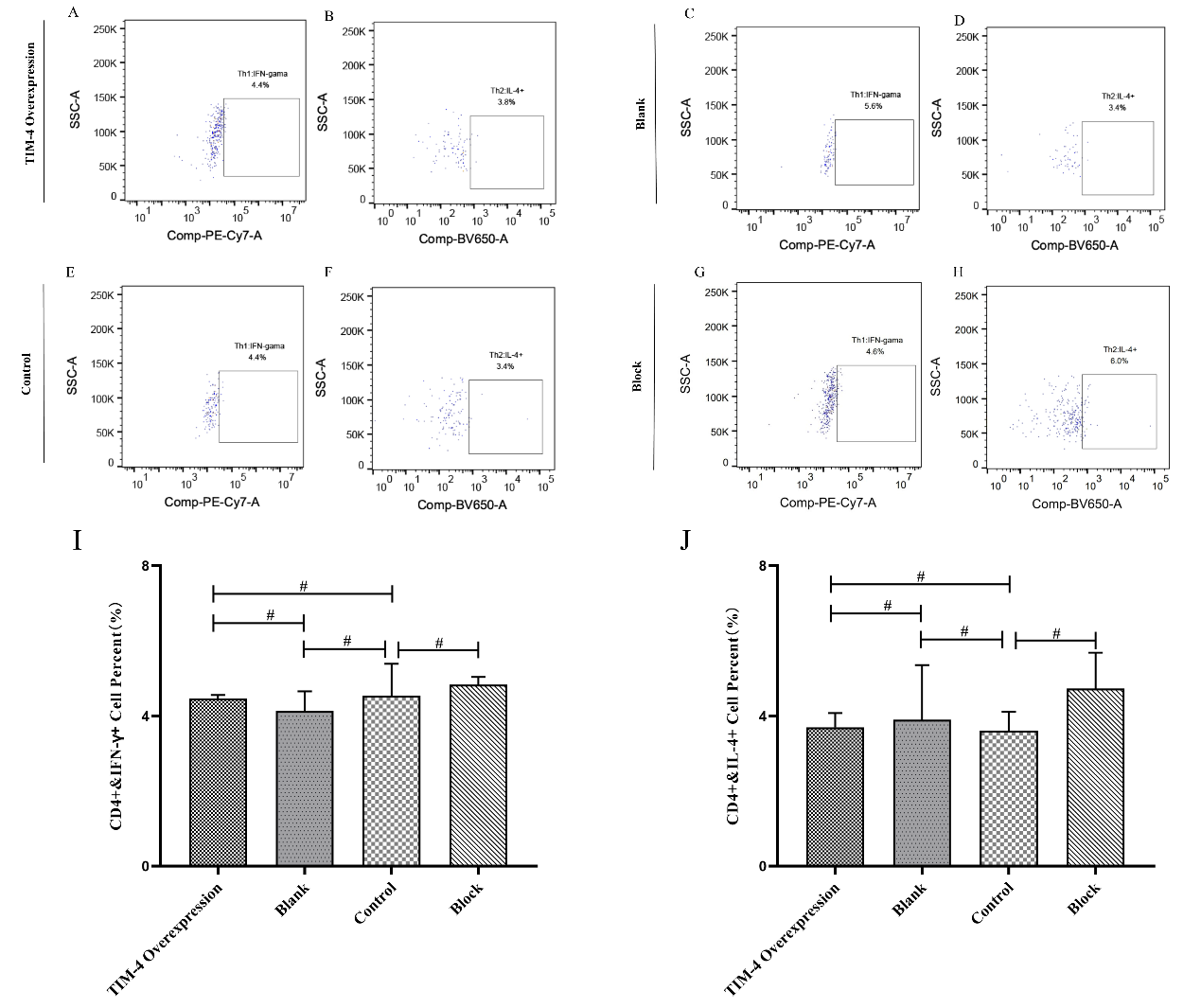


**Fig.S11.** **Flow cytometry was used to detect T lymphocyte subsets in different treatment groups**

A: Proportion of Th1 lymphocytes in TIM-4 Overexpression group; B: Proportion of Th2 in TIM-4 Overexpression group; C: Proportion of Th1 lymphocytes in Blank group; D: Proportion of Th2 in Blank group; E: Proportion of Th1 lymphocytes in Control group; F: Proportion of Th2 in Control group; G: Proportion of Th1 lymphocytes in Block group; H: Proportion of Th2 in Block group; I: Statistical results of TH1 cells in four different groups; J: Statistical results of Th2 cells in four different groups. #: no significant difference.


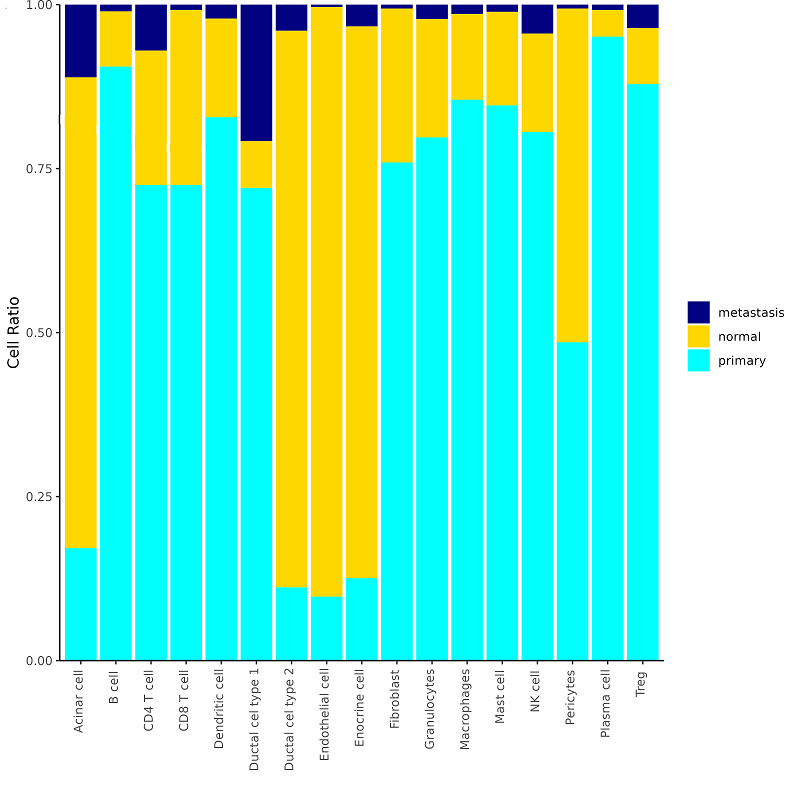


**Fig.S12.** **Single-cell sequencing data showed the proportion of various cell types in primary pancreatic cancer tissue, adjacent tissue, and metastatic tissues**


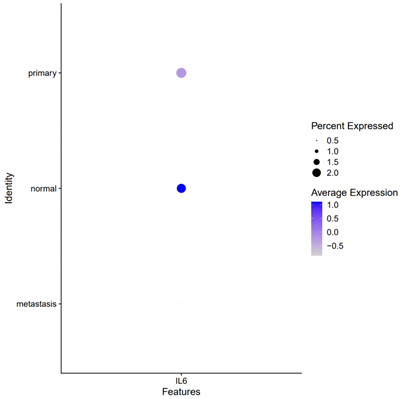


**Fig.S13.** **Single-cell sequencing data showed IL-6 expression levels in the primary pancreatic cancer tissue, adjacent tissue, and metastatic tissue**


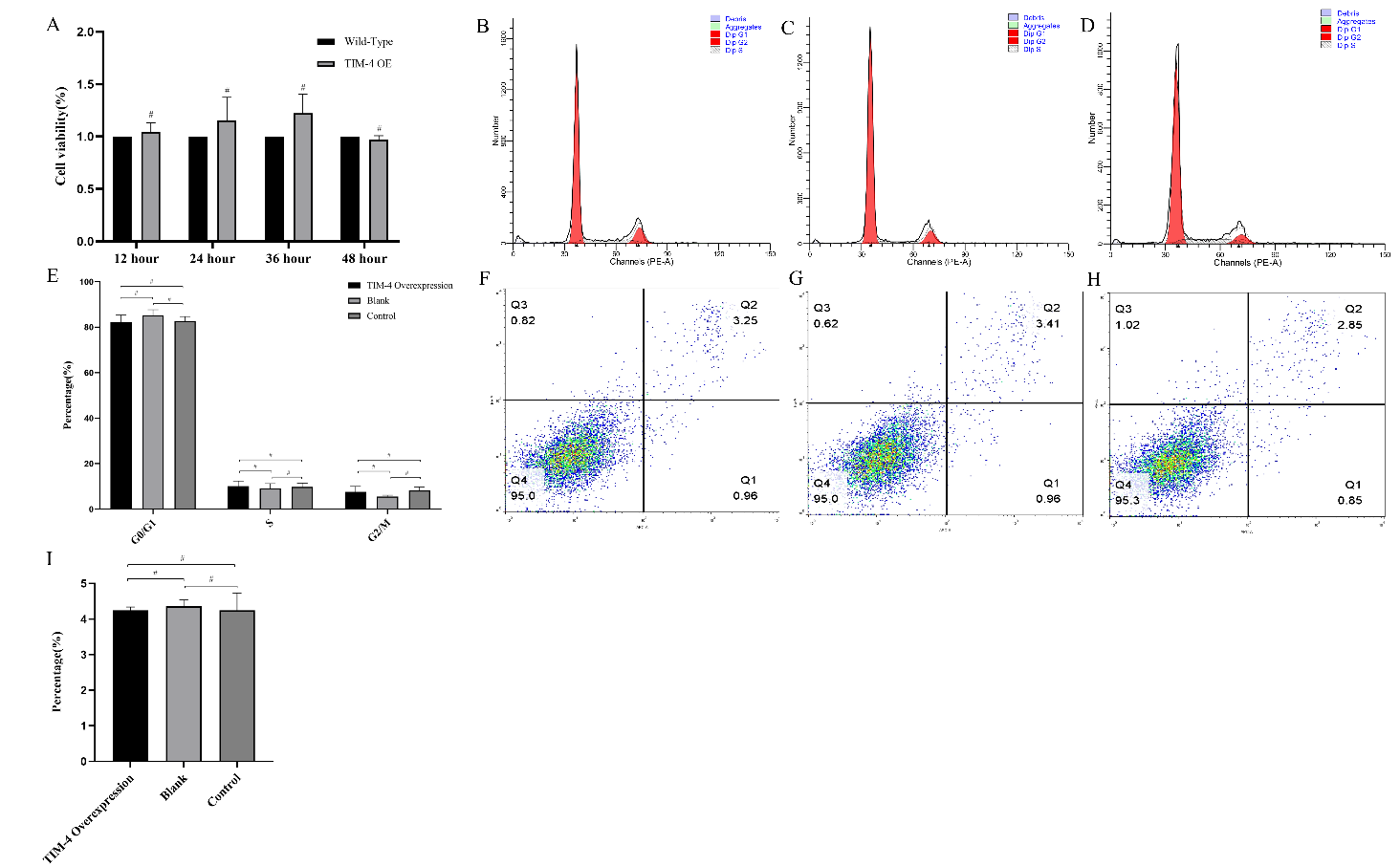


**Fig.S14. Proliferation and apoptosis of TIM-4 overexpressed Panc02 cells**

A. CCK-8 showed the proliferation of TIM-4 overexpressed in Panc02 cells and Panc02 cells. B. Cell cycle in Panc02 cells with TIM-4 overexpression; C. Cell cycle of Panc02 cells with lentivirus vector group; D. Cell cycle of wild-type Panc02 cells; E. Cell cycle statistical result of TIM-4 overexpressed Panc02 cells and control cells; F. Apoptosis of Panc02 cells with TIM-4 overexpression; G. Apoptosis of Panc02 cells with lentivirus vector group; H. Apoptosis of wild-type Panc02 cells; I. Statistical results of apoptotic cells in TIM-4 overexpressed Panc02 cells and control cells; *** : P<0.001; #: The difference was not statistically significant.

**Table S1.** **Clinical data of 30 patients with PDAC**

| No. | Gender | Age | Pathology | Tumor diameter(cm) | lymphatic metastasis | Differentiation | TNM Stage | Pre-operation CA19-9 |
| --- | --- | --- | --- | --- | --- | --- | --- | --- |
| 1 | Male | 53 | PDAC | 2.7 | No | Medium | IB | 96.7 |
| 2 | Female | 70 | PDAC | 4 | No | Medium-High | IB | >1000 |
| 3 | Male | 55 | PDAC | 2.5 | No | Medium | IB | 158 |
| 4 | Male | 57 | PDAC | 3.8 | No | Medium | IB | 362 |
| 5 | Male | 62 | PDAC | 2.7 | Yes | Medium | IIB | 119 |
| 6 | Female | 65 | PDAC | 3 | No | Medium-Low | IB | 195 |
| 7 | Male | 44 | PDAC | 3.5 | No | Low | IB | 232 |
| 8 | Female | 58 | PDAC | 2.5 | Yes | Medium | IIB | 3.95 |
| 9 | Male | 79 | PDAC | 3 | Yes | Medium-Low | IIB | 104 |
| 10 | Male | 60 | PDAC | 2.5 | No | Medium | IB | 102 |
| 11 | Male | 80 | PDAC | 2.1 | Yes | High | IIB | 307.4 |
| 12 | Female | 61 | PDAC | 3 | No | Medium-Low | IB | 550.5 |
| 13 | Male | 56 | PDAC | 3 | Yes | Medium | III | 56.35 |
| 14 | Female | 51 | PDAC | 3 | Yes | Medium-Low | IIB | 1.13 |
| 15 | Male | 60 | PDAC | 3.5 | Yes | Medium-Low | III | 280.1 |
| 16 | Female | 65 | PDAC | 6 | Yes | Medium-Low | IIB | 302 |
| 17 | Male | 72 | PDAC | 4 | No | Low | IB | 14.09 |
| 18 | Female | 49 | PDAC | 5.5 | Yes | Low | III | 145.7 |
| 19 | Male | 61 | PDAC | 1.5 | Yes | Medium-Low | IIB | 74.97 |
| 20 | Female | 77 | PDAC | 3 | Yes | Medium-Low | IIB | 401.1 |
| 21 | Female | 51 | PDAC | 5.3 | Yes | Medium-Low | IIB | >1000 |
| 22 | Female | 62 | PDAC | 4 | No | Medium-Low | IB | 1.98 |
| 23 | Male | 63 | PDAC | 4.5 | Yes | Medium-Low | III | 0.6 |
| 24 | Female | 60 | PDAC | 3.5 | No | Medium-Low | IB | >1000 |
| 25 | Female | 63 | PDAC | 3.2 | No | Medium-Low | IB | 810.7 |
| 26 | Male | 71 | PDAC | 4 | Yes | Medium-Low | III | 66.77 |
| 27 | Male | 61 | PDAC | 4 | No | Medium | IIA | 48.27 |
| 28 | Male | 57 | PDAC | 3.5 | Yes | Medium-Low | IIB | 2.76 |
| 29 | Male | 76 | PDAC | 3 | Yes | Medium-Low | IIB | 9.79 |
| 30 | Male | 46 | PDAC | 3.2 | No | Medium | IB | 43.45 |

**Table S2. List of primers**

| Gene | Species | Name | Sequence(5’→3’) |
| --- | --- | --- | --- |
| TIM-4 | Human | Forward primer | TCCGCACTGATGGAATGAGG |
|  |  | Reverse primer | CTTTCACTGGGGTTTAAGATGGT |
| TIM-4 | Mouse | Forward primer | TTGTTTCTGGCGTTTCTC |
|  |  | Reverse primer | GTCTTCATCATCCCTCCC |
| GAPDH | Human | Forward primer | GGAGCGAGATCCCTCCAAAAT |
|  |  | Reverse primer | GGCTGTTGTCATACTTCTCATGG |
| GAPDH | Mouse | Forward primer | TGACCTCAACTACATGGTCTACA |
|  |  | Reverse primer | CTTCCCATTCTCGGCCTTG |

**Table S3. List of antibodies**

| Antibody | Cat.No | Manufacturer | Manufacturer Location | Concentration |
| --- | --- | --- | --- | --- |
| Anti-TIM 4 antibody | ab47637 | Abcam | Massachusetts, USA | 1:1000 |
| GAPDH Monoclonal Antibody | 60004-1-Ig | Proteintech | Wuhan, China | 1:20000 |
| Beta Actin Monoclonal Antibody | 66009-1-Ig | Proteintech | Wuhan, China | 1:20000 |
| Goat anti-Mouse IgG Secondary Antibody | L3032 | Signalway Antibody | Maryland, USA | 1:4000 |
| Goat anti-Rabbit IgG Secondary Antibody | L3012 | Signalway Antibody | Maryland, USA | 1:4000 |

**Table S4. List of antibodies for multiplex fluorescent immunohistochemical**

| Antibody | Cat.No | Manufacturer | Manufacturer Location | Concentration |
| --- | --- | --- | --- | --- |
| Anti-TIM 4 antibody | 75484 | Cell Signaling Technology | Massachusetts, USA | 1:200 |
| Anti-Cytokeratin 19 antibody | Ab52625 | Abcam | Massachusetts, USA | 1:500 |
| Anti-CD3 antibody | ZM-0417 | ZSGB-BIO | Beijing, China | 1:50 |
| Anti-CD68 antibody | ZM-0060 | ZSGB-BIO | Beijing, China | 1:500 |
| Anti-CD163 antibody | ZM-0428 | ZSGB-BIO | Beijing, China | 1:200 |
| Anti-CD4 antibody | PA285 | ABCARTA | Suzhou, China | Ready-to-use |
| Anti-CD8 antibody | IR623 | DAKO | Shanghai, China | Ready-to-use |
| Anti-FOXP3 antibody | PA448 | ABCARTA | Suzhou, China | 1:1 |

**Table S5.** **Clinical data of 79 patients with PDAC with tissue microarray**

| Clinical features | Classification | TIM4+Ratio | | P Value |
| --- | --- | --- | --- | --- |
|  |  | Low-expression | High-expression |  |
|  |  | 48 | 31 |  |
| Age, mean (SD) |  | 58.45 (6.11) | 55.91 (10.06) | 0.401 |
| Gender, Proportion(%) |  |  |  | 0.835 |
|  | Male | 29（60.42） | 18（58.64） |  |
|  | female | 19（39.58） | 13（41.36） |  |
| Tumor size, mean (SD), cm |  | 3.56 (1.04) | 3.97 (1.12) | 0.066 |
|  |  |  |  |  |
| Differentiation, proportion (%) |  |  |  | 0.582 |
|  | High | 2 (4.17) | 0 (0) |  |
|  | Medium-High | 4 (8.33) | 1 (3.23) |  |
|  | Medium | 22 (45.83) | 17 (54.84) |  |
|  | Medium-Low | 18 (37.50) | 11 (35.48) |  |
|  | Low | 2 (4.17) | 2 (6.45) |  |
| Vascular invasion, proportion (%) |  |  |  | 0.733 |
|  | No | 26 (54.17) | 18 (58.06) |  |
|  | Yes | 22 (45.83) | 13 (41.94) |  |
| Neural invasion, proportion (%) |  |  |  | 0.696 |
|  | No | 15 (31.25) | 11 (35.48) |  |
|  | Yes | 33 (68.75) | 20 (64.52) |  |
| T Grade, proportion (%) |  |  |  | 0.172 |
|  | T1 | 5 (10.42) | 0 (0) |  |
|  | T2 | 22 (45.83) | 13 (41.94) |  |
|  | T3 | 16 (33.33) | 15 (48.39) |  |
|  | T4 | 5 (10.42) | 3 (9.68) |  |
| N Grade, proportion (%) |  |  |  | 0.707 |
|  | N0 | 17 (35.42) | 11 (35.48) |  |
|  | N1 | 23 (47.92) | 17 (54.84) |  |
|  | N2 | 8 (16.67) | 3 (9.68) |  |
| M Grade, proportion(%) |  |  |  | 0.936 |
|  | M0 | 36 (75.00) | 23 (74.19) |  |
|  | M1 | 12 (25.00) | 8 (25.81) |  |
| TNM Stage, proportion(%) |  |  |  | 0.767 |
|  | Ι/II | 31 (64.58) | 19 (61.29) |  |
|  | III/IV | 17 (35.41) | 12 (38.71) |  |
|  |  |  |  |  |

**Table S6.Target gene sequence of TIM-4 in Panc02**

atgtccaaggggcttctcctcctctggctggtgacggagctctggtggctttatctgacaccagctgcctcagaggatacaataatagggtttttgggccagccggtgactttgccttgtcattacctctcgtggtcccagagccgcaacagtatgtgctggggcaaaggttcatgtcccaattccaagtgcaatgcagagcttctccgtacagatggaacaagaatcatctccaggaagtcaacaaaatatacacttttggggaaggtccagtttggtgaagtgtccttgaccatctcaaacaccaatcgaggtgacagtggggtgtactgctgccgtatagaggtgcctggctggttcaatgatgtcaagaagaatgtgcgcttggagctgaggagagccacaacaaccaaaaaaccaacaacaaccacccggccaaccaccaccccttatgtgaccaccaccaccccagagctgcttccaacaacagtcatgaccacatctgttctcccaaccaccacaccaccccagacactagccaccactgccttcagtacagcagtgaccacgtgcccctcaacaacacctggctccttctcacaagaaaccacaaaagggtccgccttcactacagaatcagaaactctgcctgcatccaatcactctcaaagaagcatgatgaccatatctacagacatagccgtactcaggcccacaggctctaaccctgggattctcccatccacttcacagctgacgacacagaaaacaacattaacaacaagtgagtctttgcagaagacaactaaatcacatcagatcaacagcagacagaccatcttgatcattgcctgctgtgtgggatttgtgctaatggtgttattgtttctggcgtttctccttcgagggaaagtcacaggagccaactgtttgcagagacacaagaggccagacaacactgaagatagtgacagcgtcctcaatgacatgtcacacgggagggatgatgaagacgggatcttcactctctga

**Table S7. List of antibodies for flow cytometry**

| Antibody | Cat.No | Manufacturer | Manufacturer | Label |
| --- | --- | --- | --- | --- |
| Anti-TIM 4 antibody | 354004 | Biolegend | USA | PE |
| Anti-TIM 4 antibody | 564147 | BD | USA | PE |
| Anti-CD3 antibody | 557596 | BD | USA | Ms CD3e APC-Cy7 145-2C11 |
| Anti-CD4 antibody | 560350 | BD | USA | Ms CD4 BV711 GK1.5 |
| Anti-CD8 antibody | 551162 | BD | USA | Ms CD8a PerCP-Cy5.5 53-6.7 |
| Anti-CD25 antibody | 553075 | BD | USA | Ms CD25 PE 3C7 |
| Anti-FOXP3 antibody | 560401 | BD | USA | Ms Foxp3 Alexa 647 MF23 |
| Anti-IFN-γ antibody | 557649 | BD | USA | Ms IFN-γ PE-Cy7 XMG1.2 |
| Anti-IL-4 antibody | 564004 | BD | USA | Ms IL-4 BV650 11B11 |
| Anti-IL-17a antibody | 564171 | BD | USA | Ms IL-17A BV786 TC11-18H10 |
| Anti-mouse TIM-4 | BE0171 | Bioxcell | USA | Blocking antibody |

**Table S8. 30 major KEGG-pathways and their corresponding genes**

| KEGG Pathway Term ID | KEGG Pathway Term | Rich Ratio | Candidate Gene Num | Gene Symbol |
| --- | --- | --- | --- | --- |
| 4060 | Cytokine-cytokine receptor interaction | 0.037 | 11 | Csf2,Ghr,Il11,Il6,Inhbb,Cxcl2,Tnf,Gdf15,Tnfsf15,Pf4,Tnfrsf25 |
| 5323 | Rheumatoid arthritis | 0.070 | 6 | Angpt1,Csf2,Il11,Il6,Cxcl2,Tnf |
| 0534 | Glycosaminoglycan biosynthesis-heparan sulfate / heparin | 0.125 | 3 | Xylt1,Extl1,Ndst3 |
| 4640 | Hematopoietic cell lineage | 0.053 | 5 | Cd5,Csf2,Il11,Il6,Tnf |
| 5134 | Legionellosis | 0.068 | 4 | Hspa1b,Il6,Cxcl2,Tnf |
| 5332 | Graft-versus-host disease | 0.070 | 4 | H2-Q6,H2-Q4,Il6,Tnf |
| 1040 | Biosynthesis of unsaturated fatty acids | 0.094 | 4 | Psph,Cth,Pycr1,Asns |
| 4064 | NF-kappa B signaling pathway | 0.046 | 5 | Lbp,Lck,Cxcl2,Tnf,Traf1 |
| 4668 | TNF signaling pathway | 0.044 | 5 | Csf2,Il6,Cxcl2,Tnf,Traf1 |
| 1230 | Biosynthesis of amino acids | 0.052 | 4 | Psph,Cth,Pycr1,Asns |
| 5132 | Salmonella infection | 0.053 | 4 | Csf2,Il6,Lbp,Cxcl2 |
| 0450 | Selenocompound metabolism | 0.118 | 2 | Cth,Inmt |
| 4612 | Antigen processing and presentation | 0.045 | 4 | H2-Q6,H2-Q4,Hspa1b,Tnf |
| 4657 | IL-17 signaling pathway | 0.044 | 4 | Csf2,Il6,Cxcl2,Tnf |
| 5166 | Human T-cell leukemia virus 1 infection | 0.029 | 7 | H2-Q6,Csf2,Egr2,H2-Q4,Il6,Lck,Tnf |
| 4061 | Viral protein interaction with cytokine and cytokine receptor | 0.039 | 4 | Il6,Cxcl2,Tnf,Pf4 |
| 4151 | PI3K-Akt signaling pathway | 0.023 | 8 | Angpt1,Areg,Col6a1,Fgf18,Ghr,Il6,Ppp2r2c,Gng13 |
| 4630 | Jak-STAT signaling pathway | 0.030 | 5 | Csf2,Ghr,Il11,Il6,Socs2 |
| 4650 | Natural killer cell mediated cytotoxicity | 0.040 | 4 | Csf2,Lck,Tnf,Ulbp1 |
| 4931 | Insulin resistance | 0.036 | 4 | Ppp1r3e,Il6,Tnf,Trib3 |
| 5146 | Amoebiasis | 0.038 | 4 | Csf2,Il6,Cxcl2,Tnf |
| 5167 | Kaposi sarcoma-associated herpesvirus infection | 0.028 | 6 | H2-Q6,Csf2,H2-Q4,Il6,Cxcl2,Gng13 |
| 5321 | Inflammatory bowel disease (IBD) | 0.050 | 3 | Il6,Rorc,Tnf |
| 5330 | Allograft rejection | 0.049 | 3 | H2-Q6,H2-Q4,Tnf |
| 5203 | Viral carcinogenesis | 0.026 | 6 | H2-Q6,Egr2,H2-Q4,Rbpjl,Traf1,H2bc8 |
| 4940 | Type I diabetes mellitus | 0.044 | 3 | H2-Q6,H2-Q4,Tnf |
| 0062 | Fatty acid elongation | 0.069 | 2 | Elovl6,Acot2 |
| 5202 | Transcriptional misregulation in cancer | 0.028 | 5 | Csf2,Ddit3,Il6,Traf1,Nupr1 |
| 1523 | Antifolate resistance | 0.067 | 2 | Il6,Tnf |
| 5133 | Pertussis | 0.039 | 3 | C4bp,Il6,Tnf |

**Table S9. Raw statistical data of flow cytometry to detect Th1/Th2 lymphocyte subsets in different treatment groups (mean%±SD)**

|  | Th1 | Th2 |
| --- | --- | --- |
| TIM-4 Overexpression | 4.467±0.094 | 3.7±0.374 |
| Blank | 4.133±0.525 | 3.9±1.454 |
| Control | 4.533±0.858 | 3.6±0.510 |
| Block | 4.833±0.205 | 4.733±0.953 |

**Table S10. Raw statistical data of flow cytometry to detect Th17/Treg lymphocyte subsets in different treatment groups (mean%±SD)**

|  | Th17 | Treg |
| --- | --- | --- |
| TIM-4 Overexpression | 1.733±0.287 | 16.3±0.294 |
| Blank | 4.433±0.125 | 11.667±1.066 |
| Control | 4.434±0.167 | 11.767±1.184 |
| Block | 12.7±0.829 | 7.3±0.432 |

**Table S11. Raw statistical data of flow cytometry to detect CD8^+^/Treg lymphocyte subsets in different treatment groups of mouse (mean%±SD)**

|  | Treg | CD8^+^ | Co-culture CD8^+^ |
| --- | --- | --- | --- |
| TIM-4 Overexpression | 16.3±0.294 | 6.167±0.249 | 5.567±0.309 |
| Blank | 11.667±1.066 | 8.1±0.163 | 7.967±0.910 |
| Control | 11.767±1.184 | 8.2±0.356 | 7.933±0.419 |
| Block | 7.3±0.432 | 12.7±0.829 | 9.6±0.589 |
